# Artificial Intelligence Applications in Laser-Assisted Refractive Surgery—A Comprehensive Review

**DOI:** 10.3390/diagnostics16203380

**Published:** 2026-10-17

**Authors:** Paul Filip Curcă, Mioara Laura Macovei, Ovidiu Mușat, Mihail Zemba, Andreea Alexandra Mihaela Mușat, Mihai Adrian Păsărică, Silvia Victoria Prodescu, Cătălina Ioana Tătaru, Călin Petru Tătaru

**Affiliations:** 1 Ophthalmology Department, “Carol Davila” University of Medicine and Pharmacy, Central Military Emergency University Hospital “Dr. Carol Davila”, 010825 Bucharest, Romania; filipcurca@yahoo.com (P.F.C.); laura.macovei@yahoo.com (M.L.M.); mhlzmb@yahoo.com (M.Z.); calinpetrutataru@yahoo.com (C.P.T.); 2 Doctoral School, Ophthalmology Department, “Carol Davila” University of Medicine and Pharmacy, 020021 Bucharest, Romania; andreea-alexandra.musat@rez.umfcd.ro (A.A.M.M.); silviavpro@gmail.com (S.V.P.); 3 Department of Ophthalmology, Clinical Institute for Ophthalmological Emergencies “Prof. Dr. Mircea Olteanu”, 010464 Bucharest, Romania; m.pasarica@yahoo.com (M.A.P.); catalina_tataru@yahoo.com (C.I.T.); 4 Department of Ophthalmology, Alcor Clinic, 031281 Bucharest, Romania; 5 Department of Ophthalmology, Clinical Institute for Ophthalmological Emergencies “Prof. Dr. Mircea Olteanu”, Carol Davila University of Medicine and Pharmacy, 010464 Bucharest, Romania

**Keywords:** artificial intelligence, AI, machine learning, ML, refractive surgery, laser surgery, PRK, LASIK, SMILE, keratoconus

## Abstract

**Background/Objectives**: Artificial Intelligence (AI) technology—in the form of Machine Learning (ML) models and other models specialized in image recognition and processing, such as Convolutional Neural Networks Visual Transformers (CNN-ViTs)—is increasingly being used in ophthalmology. This review summarizes the main AI applications for laser-assisted refractive surgery. **Methods**: A wide-ranging literature search on the PubMed and ISI Thompson Web of Science databases was conducted. **Results**: AI is an all-encompassing technological concept that refers to either specific applications of ML/CNN models, such as in keratoconus screening or diagnosis, or agentic/multimodal AI applications that process different data types using a variety of AI models. The most clinically established applications found were corneal ectasia and keratoconus screening, in which AI models can reliably exceed 95% diagnostic accuracy, specificity, and sensitivity; nomogram-derived laser ablation programming; optimization of ray-tracing calculations for ray-traced ablation profiles; patient screening and selection for refractive surgery; visual and refractive result prediction; assessment of postoperative corneal ectasia risk; detection of intraoperative complications; research on the laser coupling effect; and other applications such as monitoring eye rubbing behavior in corneal ectasia suspects. Thus, singular, specific applications of AI models have made the largest clinical contributions. These results must be interpreted with a comparison of internal versus external validation, calibration, and generalizability. Overall the technology is highly promising. Agentic/multimodal AI frameworks are currently being discussed, with pilot studies published and applicability proposed in the context of large, high-patient-volume clinical practices. The technological advent of SHAP force plot graphs and saliency maps has improved understanding of the factors that contribute mechanistically to AI model reasoning, leading to more informed clinical decisions or research applicability, such as in finding parameters or factors linked to postoperative ectasia. **Conclusions**: AI is already established in laser-assisted refractive surgery in the form of models specialized in data-intensive analysis. At present, the best-performing novel AI models originate from the domain of large or multi-hospital settings due to requirements for multiple latest-generation diagnostic devices, high-volume patient data, computing expertise, and resources. Future directions could include clinical standardization and trickle-down dissemination of the technology. Agentic/multimodal AI has the potential to benefit large clinical/patient settings or offer access to expert knowledge in smaller practice settings. Agentic/multimodal AI implementation is offset by challenges in complexity, the technological limitations of LLMs (“hallucinations”), and ethical and legislative factors.

## 1. Introduction

Refractive surgery is the surgical correction of refractive errors, such as myopia, hyperopia, and astigmatism [1], benefiting the quality of life of the patient, working ability, and daily performance [1,2]. Ever since surgeon Jose I. Barraquer Moner conceived and developed the first stromal sculpting methods to correct refractive error (“keratomileusis”, meaning “sculpting” of the “cornea”) [3], refractive surgery has become an ever-evolving field driven by advancements in the understanding of ocular biomechanics and visual optics [1]. The advent of laser-assisted refractive surgery, such as the Photorefractive Keratomileusis (PRK) and Femtosecond Laser-Assisted In Situ Keratomileusis (FS-LASIK) techniques [2,3], revolutionized visual outcomes [3]. Further technological advances in laser technology led to improvements in the original FS-LASIK and PRK surgical techniques and the advent of new laser-assisted refractive surgery techniques such as Transepithelial Photorefractive Keratomileusis (Trans-PRK) [4] and SmartSurf^ACE^ [4]/SmartPulse^®^ Trans-PRK [5,6]; the original Small-Incision Lenticule Extraction (SMILE) technique [7]; and similar lenticle extraction variants such as Femtosecond Lenticule Extraction (FLEx) [8], Refractive Lenticule Extraction (ReLEx) [9,10], Corneal Lenticule Extraction for Advanced Refractive Correction (CLEAR) [11,12], refractive corneal lenticule extraction (RCLE) [11], Smooth Incision Lenticule Keratomileusis (SILK™) [13], and SMILE Pro [14,15]. Another revolution in refractive surgery is the advent of customized corneal ablation profiles by Kanellopoulos A.J. et al. [16] and the use of Alcon Wavelight^®^ Contoura technology, popularized by Motwani M. et al. [17,18,19], and Schwind Amaris topography-based customized Trans-PRK [20,21]. It seems increasingly likely that the next generation of refractive surgery could yet again involve software, only this time in the form of Artificial Intelligence (AI) applications. From nascent analytical models, such as Phorcides popularized by Stulting R.D. et al. [22], to current ray-tracing models (Ray-Tracing-Guided FS-LASIK) [23] and other AI technologies, this review seeks to summarize AI-assisted technological improvements in refractive surgery.

Artificial Intelligence (AI) is a revolutionary computational improvement in which artificial networks function similarly to human neuronal networks, processing data, carrying out tasks autonomously [24,25], and continuously learning so that future tasks can be better resolved. AI first requires a Machine Learning or Neural Network AI model to be trained, a process that requires large amounts of high-quality data such as clinical images and significant processing power for running the network. The training data must be standardized in a format that can be understood by the AI training software [26] and, depending on the learning method, manual annotation of the data to point out the correct result (for example, correct diagnosis) or a control group of correctly annotated data may be required [26,27,28,29]. For example, Burlina et al. and Grassman et al. used over 130,000 [30] and 120,656 fundus photographs [31], respectively, to train Deep Learning (DL) algorithms to interpret Age-related Macular Degeneration (ARMD) [30,31]. After the training was completed, the AI model was deployable and required significantly fewer resources since the model could only be loaded in memory and had sufficient resources to return query results in a reasonable timeframe.

Currently, AI models can either be accessed via online/offline cloud computing or used locally on a sufficiently powerful machine.

One of the first general Large Language Models that became accessible to run on personal computers was DeepSeek R1 [32], which could be loaded in memory to run locally on a PC provided there was sufficient Video Random Access Memory (VRAM) or combined Random Access Memory (RAM)/VRAM pooled memory available [33,34,35]. From a processing power requirement point of view, DeepSeek R1 could either be loaded in RAM and run using a Central Processing Unit (CPU) or loaded in the Graphics Processing Unit (GPU) VRAM [33,34] and run on GPU power, which is many times faster since GPUs excel at managing parallel workloads such as AI processing [33,34,35]. Mikhail D. et al. all reported good results (82%) in resolving ophthalmology-related questions (queries) using an off-the-shelf DeepSeek R1 model [36]. The 671-billion-parameter DeepSeek-R1 (compressed and dynamically quantized) still required a specialized system to run, requiring over 131 Gigabytes of VRAM/RAM to load; however, distilled (reduced-parameter; and thus reduced “smartness”) versions of the original DeepSeek-R1 [37] could fit on a single high-end consumer graphics card or combo VRAM/RAM system like an Apple Mac Mini or Mac Studio, which share a large memory pool for RAM and Graphics Processing Unit (GPU) duty. Ongoing improvements in PC technology such as the improvement of NVIDIA’s GPU Compute Unified Device Architecture (CUDA), the advent of CPU or GPU-assisting Neural Processing Units (NPUs) [35,37], and NPU-Accelerated-Processing-Unit (NPU-APU) implementations such as the Ryzen AI APU from Advanced Micro Devices (AMD) [37] have greatly increased the processing power available on a commercial PC. However, locally run (run on a single commercially available PC) distilled models are not yet a good fit in medical applications due to reductions in the smartness and accuracy of provided answers versus the original, full-parameter model [38]. Thus, in medical applications, where accuracy is critical, the principal option for running high-end medical AI models remains cloud computing [38,39,40].Cloud computing refers to large collections of computer resources housed in data centers that are made available for short- or long-term rental as an alternative to building local processing power from scratch [41]. 

Cloud computing allows “AI for all” since it effectively offloads hardware requirements to specialized systems and allows simple personal computers (PCs) to run AI queries on the latest and best available models, achieving fast results [39,40,41]. In the fast-evolving world of AI models, having cloud access to the latest and most performant AI model is advantageous. Alternatively, in the context of medical data protection and privacy, building private AI cloud infrastructure makes sense. For example, Chen C.J. et al. described the successful implementation of a secure private cloud AI computing server for use by medical personnel in a large clinical or hospital setting [42]. Gates, A. et al. analyzed the cloud-based and hybrid architecture challenges to be overcome in implementing an AI-First healthcare ecosystem [43] and highlighted the need for AI standardization in healthcare [43].

## 2. Materials and Methods

**Figure 1:**
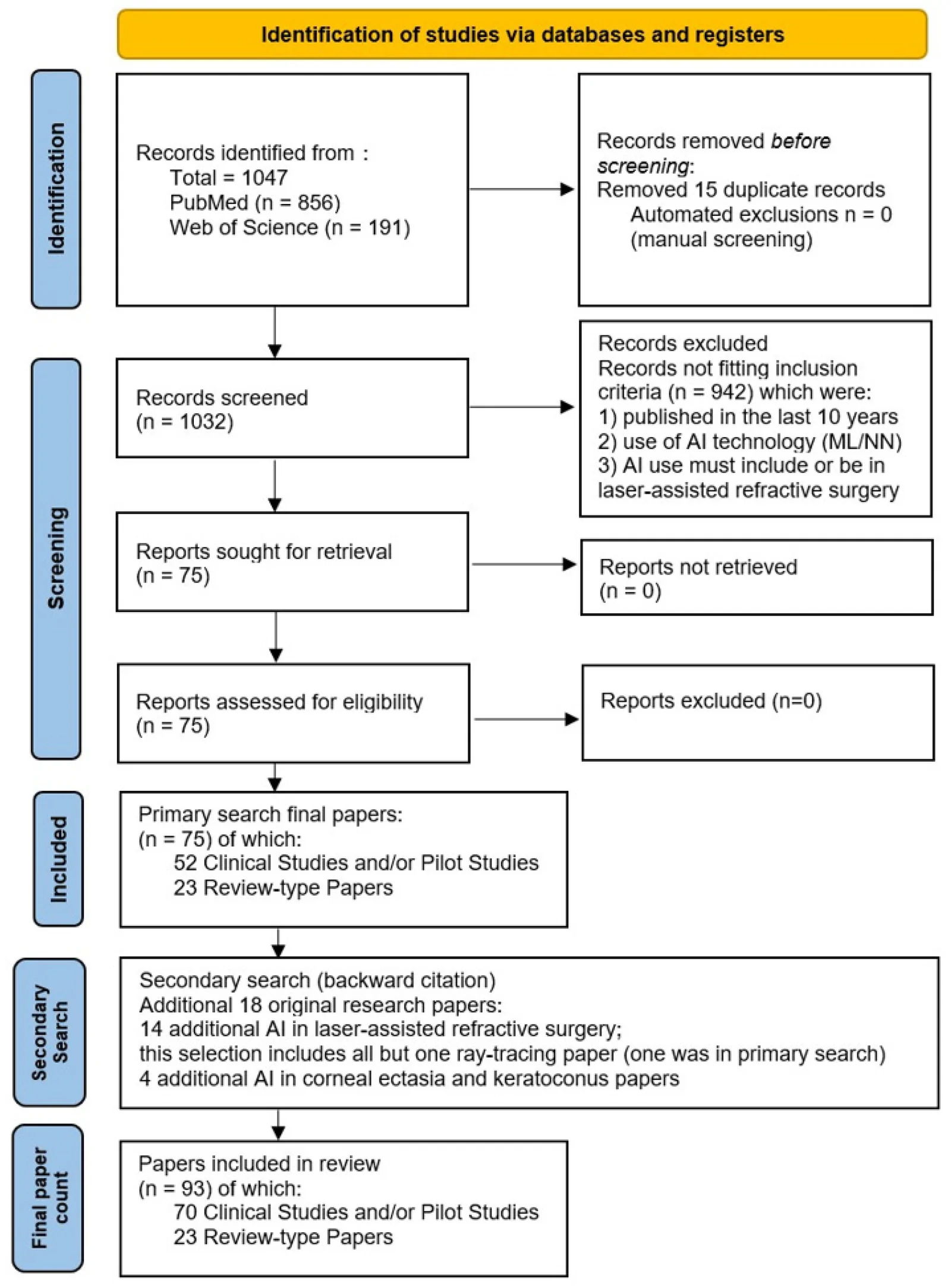
Summary of the conducted literature search. This flow diagram is adapted from the PRISMA 2020 guidelines presented by Page M.J. et al. [44]. WoS—Web of Science; AI—Artificial Intelligence; ML—Machine Learning; NN—Neural Network.

The aim of this non-systematized, wide-ranging comprehensive narrative review was to search for, select and assess literature papers focusing on Artificial Intelligence (AI) applications in laser-assisted surgery. Eligible papers were wide-ranging, including original research papers (clinical studies, pilot studies, case series reports) and reviews of any type such as narrative or systemic reviews. The authors acknowledge that this review is not Preferred Reporting Items for Systematic Reviews and Meta-Analyses (PRISMA)-compliant. For ease of presentation, we have adapted certain PRISMA elements such as the flow diagram (Figure 1), scope definition, inclusion/exclusion criteria, duplicate screening, and others.

The primary search inclusion criteria were as follows: publication in the last 10 years up to the time of writing; focus on the use of AI technology such as Machine Learning or Neural Networks; the described use of AI had to specifically include or be in laser-assisted refractive surgery. All laser-assisted refractive surgery techniques were included; for example, the most common were PRK, Trans-PRK, FS-LASIK, and SMILE.

Exclusion criteria for the primary search were as follows: papers detailing use of AI in other non-laser-assisted refractive surgery techniques such as phakic Implantable Collamer Lens (ICL) implants or Refractive Lens Exchange (RLE) surgery via premium multifocal or Extended Depth of Field (EDOF) Intraocular Lenses (IOLs).

The queried databases for the primary search were The National Library of Medicine’s PubMed and Clarivate’s Web of Science (WoS). Based on the stated inclusion and exclusion criteria, the reviewers P.F.C. and, at a later date, O.M. selected papers for inclusion. The process was performed blindly, meaning P.F.C. did not know which papers O.M. selected, and vice versa. In case of review conflict, in which one reviewer selected a paper for inclusion and the other not agreeing with this decision, the vote of the third reviewer, C.P.T., was used to settle the decision.

The primary search and review selection were performed between the end of July 2026 and the first half of August 2026. The search strategy was as follows: For PubMed, the primary search used the search term “Artificial Intelligence refractive”, which produced 856 results. For Clarivate’s Web of Science (WoS), we used the exact formulation “refractive surgery artificial intelligence”, which returned 191 results. After using WoS website search options to exclude meeting abstracts, editorial material, and proceeding papers, 172 results remained.

The search words were chosen so as to be as broad as possible while still returning a feasible-to-review number of search results; previous exploratory searches using only the term “artificial” or “artificial intelligence” returned an excessive number of results in both databases, few of which were related to ophthalmology or refractive surgery. Importantly, we mention that upon using the search terms used for the PubMed search in WoS (“artificial intelligence refractive”), an excessive number of results was returned that could not be feasibly evaluated manually without the use of automated tools; as such, for WoS, it was necessary to change the search phrase from “artificial intelligence refractive” to “refractive surgery artificial intelligence”, which returned a manageable number of results to be manually reviewed. The search was performed manually without the use of automated tools and, as such, there was no Boolean and/or selection tree used, just the stated exact search term formula.

From this initial primary search in PubMed, we identified a total of 48 relevant papers, as follows: 16 literature review papers (Table 1) and 32 clinical articles (Table 2 and Table 3). Fifteen of the clinical papers focused on AI keratoconus and corneal ectasia screening (Table 3). The remaining 17 original research articles (Table 2) focused directly on laser-assisted refractive surgery, as follows: 10 papers detailed the use of AI in improving pre/postoperative refractive calculations and laser accuracy, one was on AI and specifically studied the refractive coupling effect and Effective Optical Zone, one paper was on using AI to screen for preexisting corneal refractive surgery, two papers were on the use of Large Language Models (LLMs) and big data in patient selection for refractive surgery, two papers were on the use of AI as an educational tool in refractive surgery, and one paper was on AI use in intrastromal corneal ring surgery (a laser-assisted refractive surgery procedure, hence its inclusion).

After comparison with PubMed results and removal of duplicates, the WoS search results added seven additional review articles (Table 1), four papers detailing the use of AI in refractive surgery, one additional paper on the use of AI to screen for preexisting corneal refractive surgery, two additional papers detailing the use of big data and LLMs in patient selection for refractive surgery, six additional papers on the use of AI as an educational tool in refractive surgery, and seven additional papers on keratoconus AI screening and management (Table 3).

In total, the primary search for PubMed + WoS resulted in 75 relevant papers: 23 review papers (Table 1), 30 original research papers on AI use in performing refractive surgery (Table 2), and 22 papers on the use of AI for keratoconus and corneal ectasia (Table 3).

To further verify that this study was based on adequately selected literature, we performed a secondary manual search using information gathered from the 75 primary search papers. With regard to guidelines, the secondary search employed backward citation, as follows: upon reading the full text of the paper, one of the reviewers (P.F.C. or O.M.) noted the presence of a clinically significant study or review in the references and the decision to include it was sent to the other reviewer (P.F.C sends to O.M., and vice versa), with agreement leading to inclusion and disagreement soliciting the third opinion of C.P.T. for the final decision.

The reasoning for the secondary search was as follows. Firstly, we noticed during the primary search that older papers detailing AI model use did not use “artificial intelligence” in the tile or keywords, or use other popular terms such as “machine learning.” Secondly, we considered that papers detailing the use of AI technology together with traditional calculation formulas or algorithms could be important to include. Thus, we define the following papers identified through the secondary search: a paper assessing the impact of laser-assisted surgery, another paper on AI in ICRS, a refractive surgery screening paper, two papers detailing the use of AI models for understanding postoperative corneal ectasia and several important keratoconus AI screening and diagnosis studies not found during primary search.

The three reviewers, P.F.C., O.M., and C.P.T., discussed the inclusion of ray-tracing papers at length. While traditional ray- and path-tracing calculations are different from traditional AI techniques such as Machine Learning, AI optimization of the ray-tracing calculation process is explored by authors in several papers selected in the review. Unfortunately, to the best of our knowledge, the Alcon InnovEyes ray-tracing algorithm is proprietary and, thus, the exact extent of AI integration into the calculations is not available and cannot be stated in this paper. Due to the clinical results and importance of this novel way to calculate the ablation (via ray-tracing) we have decided to include these papers together with a disclaimer that traditional pathway- and ray-tracing (forward or reverse) is not an AI application; however, this computation-intensive process could benefit from AI optimization and, as such, we take in good faith the presence of AI optimization into this algorithm, as stated in other papers and the technology’s producer itself.

Duplicate screening was performed manually in two steps: first, each of the two reviewers, P.F.C. and O.M., independently screened their selection for duplicates. Afterwards, C.P.T. verified the work of P.F.C. and O.M. In total, 15 duplicate records were excluded by both P.F.C. and O.M., with C.P.T. providing verification of no more duplicates.

Data were extracted manually without the use of automated tools by accessing the papers’ abstracts and full text.

In total, after the secondary search, 23 review papers (Table 1), 44 original research papers on AI models in laser-assisted refractive surgery (Table 2), and 26 original research papers on AI models in corneal ectasia and keratoconus screening and diagnosis (Table 3) were selected.

## 3. Results

### 3.1. Summary Timeline of Advancements to Currently Used Artificial Intelligence Models

This subchapter presents a detailed timeline of the advancements in AI technologies which resulted in the current models used in refractive surgery. For results specific to refractive surgery, please skip to Section 3.2.

#### 3.1.1. The Concept of Machine Learning (ML)

According to Choi R.Y., Machine Learning (ML) is a field that focuses on the learning aspect of AI [139]. In classical programming, the computer is supplied with the data to be processed (dataset) and an algorithm with instructions on how to operate on the provided dataset [139]; after running the dataset through the algorithm, the computer provides a result (output) [139]. In Machine Learning, however, the computer is provided with the dataset and the end result (output) and needs to learn and generate an algorithm that best describes the relationship between the provided dataset and the expected final output [139]. Thus, in effect, in ML, the computer learns how to process the data based on the initial data provided and the expected end result.

#### 3.1.2. Training Methods Employed in Machine Learning

Machine Learning uses the following four common learning methods: supervised, unsupervised, semi-supervised, and reinforcement learning [33].

##### Supervised Learning and Resultant Neural Networks (NNs)

In supervised learning, the machine learns from a set of “labeled” training data. An ophthalmology example would be Optical Coherence Tomography (OCT) images labeled with the correct diagnosis [139,140,141]. There are many algorithms for supervised learning, with the most common in medical papers being Random Forests, Gradient-Boosted Decision Tree (GBDT), Support Vector Machines (SVMs), and Neural Networks (NNs) [141]. Random Forests use a multitude of decision trees created in parallel using randomly selected data for each and can handle complex problems faster than linear models [141]. GBDT uses multiple decision trees created in sequence: the first decision tree produces an initial prediction, the second tree predicts residual or errors of the first tree by creating another decision tree, and the process repeats until residuals converge to 0 or the number of iterations (creation of additional decision trees) reaches a specified number [141,142]. SVMs classify data based on a boundary based on given values [141].

Perceptrons and Neural Networks imitate the function of human neurons [141]. A perceptron adds weight to the input data and passes the data sequentially through a node or neuron and then passes the resultant value to an activation function, which outputs a predictive value when the sum of given values exceeds a certain threshold [140]. The model learns by comparing the output value to the actual outcome (the right answer) and then updating the weights used for the data and repeating the process [141]. A Neural Network is a multi-layered combination of perceptrons that contains an input later, multiple hidden neuron layers, and an output layer; the number of hidden neuron layers is a parameter that determines the complexity of the model and thus its processing power requirements, and is set before training the model [141]. The NN learns via backpropagation, by passing the total error obtained from the current output backwards to the preceding nodes in the hidden layer and the input layer; the model then readjusts the data weights and restarts the process, again passing data through the model [141] and learning via backpropagation. Deep Learning architectures are advanced NNs that have multiple node/neuron layers organized in a deeply nested network [141,142,143].

##### Unsupervised Learning

In unsupervised learning, the model learns from data that do not have an expected correct output; instead, the model focuses on grouping data based on their characteristics, detecting patterns in the data without having an expected correct answer [140]. Unsupervised learning is further divided into the following two types: clustering and association [140]. According to Kufel J. et al., clustering groups data based on their similarities and differences, while association analyzes the relationships between different parts of the data [140].

##### Semi-Supervised Learning

Semi-supervised is a learning paradigm that combines the use of annotated data and unlabeled data to achieve a satisfactory performance in an annotation-efficient manner [144,145]. Semi-supervised classification aims to train a classifier from both labeled and unlabeled data, so that it is better than the supervised classifier trained only on the labeled data [146]. Semi-supervised learning, in effect, uses the principles of “Consistency Regularization,” which state (according to Tarvainen A. et al.) that when a percept is changed slightly, a human typically still considers it to be the same object [146]. Correspondingly, a classification model should favor functions that give a consistent output for similar data points [146]. An example of Consistency Regularization would be rotating an OCT image by 5° degrees; while the image has been slightly altered versus the original via rotation, it still represents the original labeled diagnosis and, from an AI model standpoint, the model should correctly recognize it as it recognized the original image. Thus, input images can be altered from the original by adding “noise” [146], with the goal of training a model that is more accurate and resilient to image variation.

##### AI Training Methods in Semi-Supervised Learning

The Mean Teacher (MT) method is used in object detection (identifying objects in images), an essential task in computer vision [147]. Mean Teacher aims to achieve consistency by minimizing the mean squared error between the outputs from a student model and the exponentially moving average of the students’ weights—the teacher model [144]. The model assumes a dual role as a teacher and a student: as the student, it learns as before; as the teacher, it generates targets which are then used by itself as a student for learning [146]. Tarvainen A. et al. further added that if too much weight is given to the generated targets, the cost of inconsistency outweighs that of misclassification, preventing the learning of new information and leading the model to suffer from confirmation bias; improving the quality of generated targets would mitigate this [146].

Cross Pseudo Supervision (CPS) trains two models simultaneously in a supervised manner, with each model using the other’s output from the labeled and unlabeled data as an additional reference (ground truth) besides the annotations provided to the individual model [144].

Interpolation consistency training (ICT) enforces consistency between the linear combination of two predictions from two images and the prediction of a linear combination of the same two images [144].

#### 3.1.3. Deep Learning—A Technological Leap Forward in Neural Network Performance

A Deep Learning (DL) architecture is an advanced type of Neural Network (NN) that has multiple node/neuron layers organized in a deeply nested network [141,143]. According to LeCun Y. et al., Deep Learning models utilize a hierarchical learning process, where each successive layer uses the output from the previous layer as input [143]. For example, when identifying an OCT image, a Deep Learning model might begin with the input OCT image and pass it through the following series of hidden layers that learn hierarchical features [143,148]: the first layer could detect basic image information such as brightness, texture, and edges; intermediate layers could detect finer features such as retinal blood vessels and the optic disk boundary; and the deep layers could identify disease-specific patterns, estimate a diagnosis, and predict disease progression. According to LeCun Y. et al., higher layers represent more abstract aspects of the input [143].

##### Saliency Maps—Understanding How Deep Learning AI Models Think

Exact visualizations of the inner workings of a Deep Learning model are not entirely possible and are limited to the 1st layer since afterwards the information is processed in the neural network neurons, which is inherently a black box in which we cannot see clearly [149]. Instead of using exact visualization, we can track the way the model works. In order to determine the activity of intermediate layers, a reverse engineering method must be used which maps back the activities of the network towards the initial input pixel space [149]. Zeiler M.D. et al. attached a Deconvolutional Network (Decovnet) to each of the layers of their original Convolutional Neural Network (CNN) [149]; the Decovnet tracked the activity of each layer in the CNN. Zeiler M.D. et al. set all other activations in the layer to zero and passed the feature maps as input to the attached Decovnet layer, successively unpooling, rectifying, and filtering to reconstruct the activity in the layer beneath that gave rise to the chosen activation [149]. The resultant feature activation map is now called a “saliency map” [149,150], which can be generated using gradient-based visualization [150]. Saliency maps show the contribution that different regions of an image or individual pixels have in the decision-making capability of a Deep Learning AI model and thus can be extremely helpful to ophthalmologists checking on the reported result of the AI model [150]. In effect, a saliency map is a visual representation of how an AI model thinks and interprets data and helps an ophthalmologist interpret the resulting diagnosis.

##### SHapley Additive exPlanations and Force Plot Graphs—Saliency for Multimodal ML Models

Using the SHapley Additive exPlanations (SHAP) technique and force plot graphs [102], Yoo T.K. et al. investigated the decision-making process of a ML AI model [102]. SHAP provides a post hoc association and interpretation by calculating the contribution of each input variable to each decision made by a Machine Learning model, with the SHAP value providing a measure of additive feature attributions [102]. This method presents some similarities in function to a Decovnet, except it is for ML and multimodal ML models. The SHAP package, developed by Lundberg et al. [151], computes the SHAP values of each prediction case with local interpretability and represents the results graphically via a force plot [102,151]. In a force plot, red arrows show feature effects that drive the model results higher while blue arrows show feature effects that drive the model lower [102]. In effect, the force plot shows what parameters/data push the model towards option A (the Yoo T.K. et al. example recommends SMILE) in lieu of option B (recommending LASEK surgery) [102]. Thus, force plots can effectively explain ML model A versus B decision-making.

#### 3.1.4. Deep Learning Models Specialized in Ophthalmological Image Recognition

##### Convolutional Neural Networks (CNNs)

Convolutional Neural Networks are a class of Neural Networks (NNs) in Machine Learning that are specialized in solving computer vision problems by classifying visual data and identifying patterns and features in the visual data [152,153]. CNNs are NNs that use convolution in place of general matrix multiplication in at least one of their layers [154]. CNNs outperform NNs because, instead of connecting every pixel to a dedicated neuron as a NN, CNNs examine only a small neighborhood of pixels at a time using convolution, a much more efficient process [154].

Yamashita R. et al. explained the following terms for the training process of a CNN: a “parameter”/“weight” is a variable learned by the network during the training process, a “hyperparameter” is a variable set before starting training, and a “kernel” represents sets of learnable parameters applied in the operations of the CNN [153]. CNNs are inspired by the organization of the visual cortex and are adept at processing data with a grid pattern, such as images which are composed of pixels, and each pixel is stored in a two-dimensional (2D) grid as an array of numbers [153]. At its simplest, a CNN is a mathematical construct of three building block layers: convolution, pooling, and fully connected layers [153]. The convolution and pooling layers perform feature extraction [153]. A feature is any measurable visual characteristic or pattern in an image that helps distinguish one object or one classification from another [154]. A feature is not a user-set attribute or information; instead, it is derived by the Neural Network (NN) itself during the learning process and used by the NN to “distinguish” data [154]. The fully connected layer maps the extracted features into the final output such as a classification or diagnosis [153]. An input tensor is the mathematical representation of the input data introduced into a DL/CNN model [154]. Since a vector is a unidimensional (1D) tensor and a pixel is stored in a two-dimensional (2D) matrix grid as an array of numbers, then an image is most appropriately represented by a three-dimensional array of numbers arranged on a regular grid with a variable number of axes (a three-dimensional tensor, 3D tensor) [154]. Modern CNNs use 3D input tensors.

The name CNN comes from the convolution layer, which performs a convolution—a specialized type of linear operation [153] that applies a kernel (a small matrix of learnable weights/parameters) across an input image to detect specific visual patterns; the convolution operation examines local image regions individually instead of the whole image at once [154]. The convolution layer is defined by a series of hyperparameters (values that can be specified by the user in the training process) such as kernel size, number of kernels, stride, padding, and activation function [153]. As presented by Yamashita, R. et al., the kernel is applied over the input tensor, resulting in an output called a feature map [153]. Padding comprises adding zero values in rows and columns on each side of the input tensor in order to fit the center of a kernel on the outermost element and keep the same in-plane dimension [152]. Zero padding allows modern CNNs to apply more layers; otherwise, without zero padding, each successive feature map would become smaller after the convolution operation [153]. After the data have passed through the convolution layer, they move to the pooling layer.

The pooling layer downsamples the data; according to Yamashita, R. et al., it reduces the in-plane dimensionality of feature maps in order to introduce a translation invariance to small shifts and distortions, and decreases the number of subsequent learnable parameters [153], making the model more efficient to train and run. The most popular form of pooling is max pooling, which extracts patches from input feature maps and outputs only the maximum value in each feature map patch, thereby discarding all other values [153]. The hyperparameters (user-defined values) for the pooling layer are pooling method, filter size, stride, and padding [153].

The fully connected layer functions as a Neural Network of dense layers in which every input is connected to every output by a learnable weight [153]. For example, if the convolution and pooling layers extracted 512 features and the fully connected layer has 200 neurons, then each of the extracted 512 features connects to every one of the 200 neurons [154]. The fully connected layer integrates all the features previously extracted and uses them to make the final prediction; thus, in effect, it functions as a classifier [143,153,154].

An activation function (AF) is applied to the last fully connected layer [58]. The primary role of the activation function is to introduce nonlinearity into the Neural Network, enabling it to learn complex relationships [143,155]. Activation functions can also be used inside the model before the fully connected layer [143,154,155]. AFs directly affect the training and performance of a model and are divided into two categories: linear and nonlinear functions [155]. The task of the AF is to help the CNN learn the abstract properties of data through nonlinear modification [155]. Two of the most common AFs encountered in CNNs are Rectified Linear Unit (ReLU) and Softmax [155].

##### Generative Adversarial Networks (GANs)

Generative Adversarial Networks are a type of Deep Learning Neural Network developed by Ian Goodfellow [156,157,158], which can synthesize medical images by learning the mapping function from an arbitrary distribution [145]. GANs can generate realistic synthetic images and have applications in image augmentation, denoising, super-resolution, and segmentation [156]. GANs can be used to additionally process images, improving Deep Learning models [156].

A GAN architecture consists of two separate Deep Learning models—a generator which synthesizes candidate samples based on the data distribution of the original dataset, and a discriminator which tries to distinguish the synthesized candidate sample from the real sample included in the original dataset [156]. The two Deep Learning models are trained simultaneously [157,158]; rather than learning directly from real images, the generator learns from the discriminator’s feedback, adjusting its parameters to create images that are more likely to be classified as real. Hence, the discriminator continuously becomes a better classifier, while the generator continuously becomes a better image synthesizer [157,158]. The gradient information is backpropagated to the generator to improve realistic image synthesis and to the discriminator to increase real/fake discriminating capabilities [156]. GANs are used in processing retinal images, OCT, and retinal vessel segmentation [156].

A CycleGAN generates images without using matching paired images [156,159]. CycleGAN simultaneously trains two generators and two discriminators [156] and adopts a cycle consistency, allowing it to learn the characteristics of the two image domains to transfer the domains without any paired datasets [156]. Yoo, T. K et al. used a CycleGAN to transform Ultra-widefield Fundus Photography (UWFP) images into Traditional Fundus Photography (TFP) without generating fake features in the output images [160]. Another use for CycleGANs is reducing image artifacts in retinal imaging, successfully reducing overall haze, lashes, arcs, and uneven illumination [161].

#### 3.1.5. Large Language Models

According to Zhang, Z. et al., Large Language Models (LLMs) are transformer-based models [46,162,163] that are trained on massive amounts of text to learn general-purpose representations of language at scale [46]. According to Krakauer D.C. et al., the term “emergence” (referring to “emergent abilities”) for LLMs has been increasingly used to describe technological progress in prediction, problem solving, generalization, and the breaking of self-similarity with ever increasing dataset and model size [163]. Krakauer D.C. et al. argued that emergent capabilities should be distinguished from emergent intelligence [163]. While intelligence is a feature of human reasoning and language, LLMs’ emergent capabilities refer to surpassing human capability in data processing, similar to a calculator reaching a calculation result faster than a human can [163] with pen and paper. One of the most popular LLMs is Open AI’s Chat GPT [164]. Gallifant, J. et al. reported that GPT-4 is a transformer-based model pretrained to predict the next token in a document with a wide range of applications including high-risk settings like healthcare [164]. Zhao, W.X. et al. explained that a token is a small unit of text that a LLM treats as a single symbol during processing; text is first tokenized into discrete units before being processed by transformer models [165]. LLMs have improved with the advent of Reinforcement Learning from Human Feedback (RLHF) whereas fine-tuning with human feedback improves model performance and answer authenticity [166]. Anthropic’s Claude is a family of transformer-based LLMs trained using Constitutional AI [167]—an alignment framework in which the model learns to follow an explicit set of guiding principles via supervised learning and Reinforcement Learning from AI feedback (RLAIF) [167], rather than relying exclusively on Reinforcement Learning from Human Feedback (RLHF) [167,168]. This guiding principle remains the main technical and reasoning difference between Chat GPT and Claude, with Claude following a publicly available “Constitution” and requiring reduced human annotation due to RLAIF, while Chat GPT is based on RLHF to better produce human-like texts [166,168,169].

#### 3.1.6. Agentic AI

Agentic AI builds on top of Generative AI such as LLMs [170]. Agentic AI is the use of autonomous AI “agents” to perform repetitive tasks autonomously, such as data sorting and processing. Collaco, B.G. et al. reported that Agentic AI represents a promising evolution of AI for use in healthcare with systems capable of operating autonomously to achieve defined clinical goals [171]. AI agents require minimal human intervention and can integrate effectively with specialized models, often operating within multi-agent frameworks and invoking the use of external software programs or tools [171]. Furthermore, multiple AI agents can coordinate to manage tasks collaboratively [171]. Most of these systems integrate LLMs or multimodal LLMs with domain-specific models to improve task specificity [171]. Agentic AI systems are capable of pursuing specific goals autonomously, leveraging advanced reasoning techniques such as Chain-of-Thought, ReAct, and Tree-of-Thought [171]. AI agents require manual or automatic action initiation, decision-making and tool invocation such as calling upon external software through the Application Programming Interfaces (APIs).

Agentic AI presents next-generation applications for ophthalmological care. Grzybowski, A. et al. highlighted the potential for diagnosis assistance and population health screening [172]. Soetikno, B. T. emphasized the need for careful consideration of ethical issues, including authorship attribution, data privacy, bias mitigation and accountability [173]. Zhao K. et al. reported on AgentEYE, an auditable multimodal agent that integrates an LLM reasoning core with ophthalmic imaging tools, guideline retrieval and web search [174]. AgentEYE functioned as a clinician-supervised ophthalmic assistant, outperforming traditional standalone LLMs [174], suggesting a robust decision-layer orchestration [174]. Possible future applications of Agentic AI include large-scale ophthalmological screening and diagnosis assistance, and perhaps specialized data and image interpretation and processing.

### 3.2. Applications of AI in Laser-Assisted Refractive Surgery

**Figure 2:**
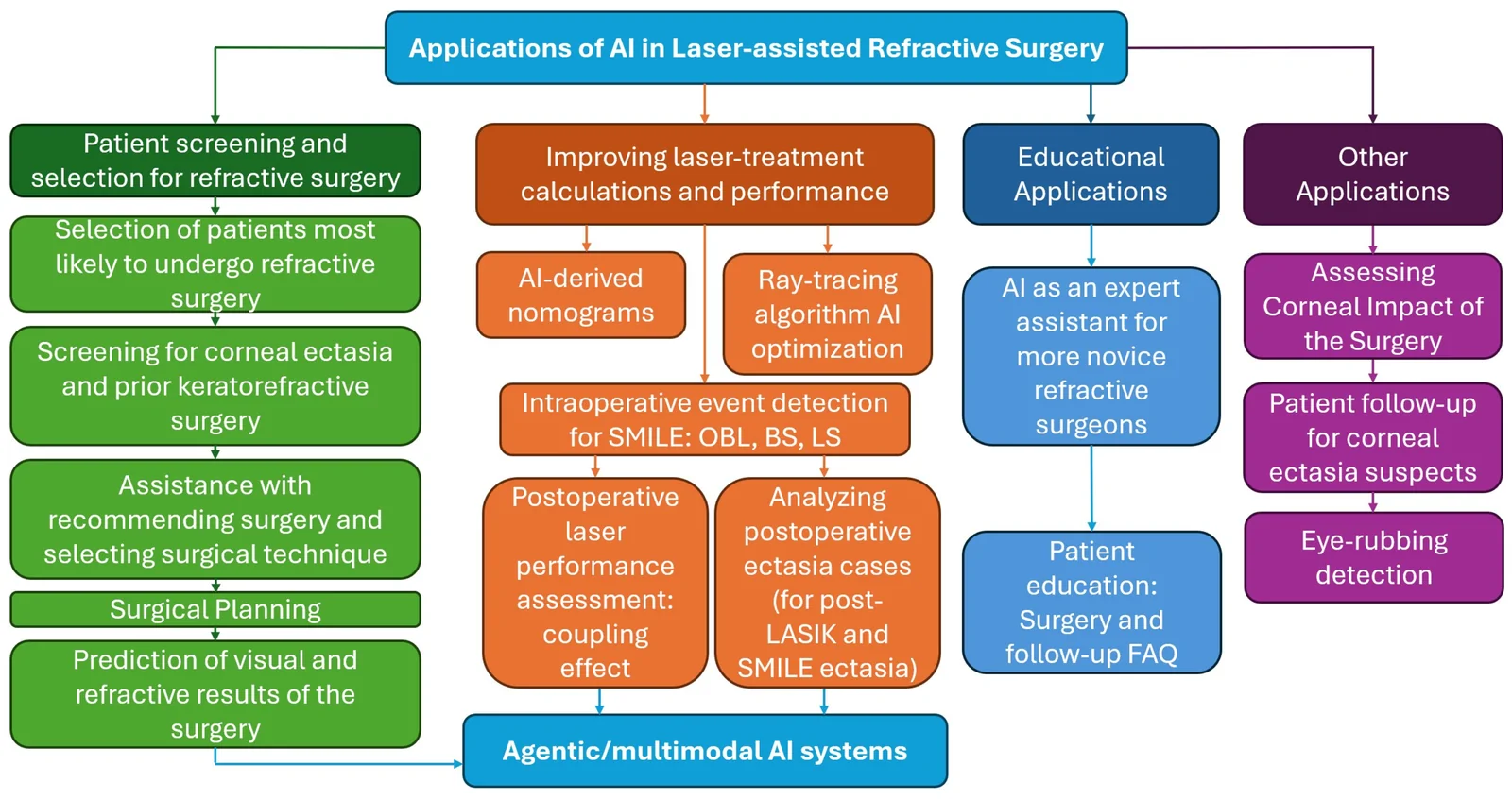
Summary of the most common applications of Artificial Intelligence technology in laser-assisted refractive surgery. AI—Artificial Intelligence; SMILE—Small-Incision Lenticule Extraction; OBL—opaque bubble layer; BS—black spots; LS—Loss of Suction; SMILE—Laser-Assisted In Situ Keratomileusis; FAQ—Frequently Asked Questions.

Figure 2 presents a summary of the most common applications of AI technology in laser-assisted refractive surgery.

#### 3.2.1. Patient Screening and Selection for Refractive Surgery

Artificial Intelligence (AI) can aid in selecting patients for refractive surgery and in recommending a therapeutic approach to be verified by a senior refractive surgeon. Since AI excels at handling large data loads, this application is especially useful in large surgical centers or centers that train and employ novice or resident refractive surgeons.

Yoo T.K. et al. carried out a study on 10,561 patients using five Machine Learning (ML) algorithms and an ensemble classifier to predict candidates that later underwent refractive surgery (10,561 training, 2640 internal validation, 5279 external validation) [103]. They reported that a Random Forest (RF) model was the single best discriminator of the algorithms tested [103]. Furthermore, the ensemble method combining all techniques led to internal accuracy of 94.1% and Area Under the Curve (AUC) of 0.983, and external accuracy of 93.4%, AUC 0.972 [103]. The paper further details prediction performance in high-risk subgroups such as high myopia (ensemble AUC 0.957), high astigmatism (ensemble AUC 0.977) and thin central corneal thickness (ensemble AUC 0.950) [103]. Errors can occur as with any AI technology, with a propensity for niche cases such as misclassification in forme fruste keratoconus, Avellino corneal dystrophy and suspected Fuchs corneal dystrophy [103]. Other times Yoo T.K. et al. reported that measurement errors in pachymetry can lead to misclassification, independent of the AI model’s performance [103]. Overall, on large data sets (large surgical practice, high patient volume, fully digital patient records), the performance of AI models can reach similar levels to that of experts, including in high-risk subgroups [103]. Yoo T.K. et al. discussed how computer-aided decision could potentially eliminate inter-clinician variability in selecting candidates for surgery and result in fewer missed contraindication cases [103]. The caveat is that the AI model itself is trained on particular data, such as the decision of certain expert ophthalmologists, and as such, bias can occur in the model [103].

In a follow-up study, Yoo T.K. et al. used an ML XGBoost algorithm to select the surgical technique for 18,480 patients from Laser Epithelial Keratomileusis (LASEK), LASIK SMILE or contraindication to refractive surgery [102]. In total, 4893 patients were selected for LASEK, 6123 for LASIK, and 5834 for SMILE, with 1630 remaining contraindicated to refractive surgery [102]. Yoo T.K. et al. reported 81% accuracy in the internal validation set and 78.9% in the external validation set [102]. The authors also detailed the decision-making process of the model using the SHapley Additive exPlanations (SHAP), using a SMILE patient as an example: the model considered the patient’s keratometric power of the posterior corneal surface inside a safe range, and their anticipation for SMILE surgery [102]; in another case, the model again predicted patient anticipation for SMILE, safe keratometric power of the posterior corneal surface and normal corneal thickness [102]. This study is notable for creating an understandable framework for the decision-making process, which could represent a crucial innovation in the use of AI models in refractive surgery. This was possible due to the use of SHAP and interpretation via force plots (Figures 6 and 7 from Yoo T.K. et al. [102]). In our opinion, a critical pillar in the future use of AI technology should be an understanding of the reasoning behind the decision, since this as valuable—or even more so—than the end result decision itself. In this way, the AI assists the refractive surgeon to the best of its ability, which could be invaluable to less-experienced refractive surgeons or residents in training.

Li J. et al. conducted a single-center ML study choosing the type of refractive surgery for 7081 myopic patients. This study is notable for using several important methods [101]. Firstly, data were pre-analyzed and curated into a more standardized format to be read by the AI model. The data were thus cleaned, missing data samples were removed, top features were selected with importance greater than 1.4%, and data imbalance was resolved using Synthetic Minority Over-Sampling Technique (SMOTE) [101]. Secondly, expert knowledge weight was added into the AI Model in the form of hand-crafted rules (e.g., PRK is more suitable for patients with thin corneas and high refractive power; do not recommend SMILE in cases with high astigmatism or irregular corneas; and avoid LASIK in cases of severe dry eye disease) [101]. Thirdly, a 10-fold cross-validation and grid search was used to train the models and determine the optimal hyperparameters to improve model performance [101]. The study evaluated six ML models and reported the best results with the RF model, with agreement with senior ophthalmologist decisions reaching as high as 0.887, and a Macro F1 of 0.8019 [101]. The best training method reported was selecting 12 features with an importance greater than 1.4%, setting max-depth to 10 and training the RF model through SMOTE [101]. Overall, the data improvement methods used in this study aided in reaching better AI model results.

#### 3.2.2. Corneal Ectasia and Keratoconus Screening for Refractive Surgery

One of the most important pathologies screened for before refractive surgery is corneal ectasia. Keratoconus is a progressive corneal ectasia characterized by stromal thinning and conical protrusion [113,114]. Early keratoconus diagnosis is key to effective visual rehabilitation and management using refractive surgery.

In the primary article search, early keratoconus detection and detecting patients at risk for corneal ectasia were by far the subjects with the most published AI clinical research. In total, 26 papers with AI models were published on clinical research [58,114,115,116,117,118,119,120,121,122,123,124,125,126,127,128,129,130,131,132,133,134,135,136,137,138] and 10 review papers were identified [59,61,63,64,65,66,67,68,69,70]. AI models are exceptionally well suited for corneal ectasia screening, with performance comparable to or exceeding expert ophthalmologists (Table 4). In fact, we consider that corneal ectasia screening and keratoconus diagnosis are applications where AI use is potentially revolutionary for clinical ophthalmological practice. AI models that integrate topographical and biomechanical data routinely offer sensitivity and specificity of diagnosis above 90%, with some AI models [121,123,126,127,133] reporting up to 95–100% specificity/sensitivity (Table 4), including Tomographic and Biomechanical Index (TBIv1) 100% specificity/sensitivity at a 0.7 cutoff [133], TBIv2 98.5% sensitivity and 98.6% specificity [124], and SCORE AI 100% specificity for keratoconus [121]. Mourgues, E. et al. published RF and LR AI models with 100% specificity on trained Tomey CASIA 2 imaging [123]; Kundu G. et al. constructed an RF AI model trained on CSO MS-39 imaging, achieving 99.1% accuracy [125]; and Du K. et al.’s large 2025 study achieved important AUC results (0.991 for subclinical keratoconus and a perfect AUC of 1.00 for keratoconus) [120].

The following paragraph presents a chronological list of important studies applying AI models to keratoconus (KC) and corneal ectasia screening; the performance values are presented in Table 4. Maeda N. et al. published pioneering work in 1995 which reported the results of an ML in keratoconus diagnosis using topographic corneal maps [138]. Chastang P.J. et al. pioneered the first ML keratoconus study using images from videokeratoscopy and a decision tree classifier [137]. The next corneal topography AI keratoconus study followed in 2005: Twa M.D. et al. used a decision tree classifier with the C4.5 algorithm, reporting a high AUC of 0.97 and 92% accuracy [136]. The first NN study, to our knowledge, was that of Saad A. & Gatinel D. in 2010, which compared FFKC characteristics with KC [135]. One of the first studies to combine Oculus Pentacam (Oculus Optikgeräte GmbH, Wetzlar, Germany) imaging and Oculus Corvis ST (Oculus Optikgeräte GmbH, Wetzlar, Germany) biomechanical measurements with AI was that of Ambrósio R Jr. et al. in 2017 [133], which recorded a Traumatic and Biomechanical Index (TBIv1) with 100% specificity and sensitivity at a cutoff value of 0.7 for detecting ectasia (keratoconus, Very Asymmetric Ectasia (VAE-E) and Fellow Eyes of VAE-E with Normal Topography (VAE-NT)) [133]. A follow-up study by Ambrósio, R. et al. published the results from the upgraded TBIv2, which presented 98.5% sensitivity and 98.6% specificity for keratoconus (KC) [126].

Other important larger studies include the following: a recent large study by Du K. et al. (2025), which reported an AUC of 0.991 for subclinical keratoconus and a perfect AUC of 1.00 for keratoconus [120]; Quanchareonsap W. et al. published a study on DL in 2024, which reported an AI Model accuracy of 95.6%, sensitivity of 93%, and specificity of 94.3% [124]; Raju R.S. et al. published a SCORE AI model with 100% specificity for keratoconus [121]; Mourgues, E. et al. published RF and LR AI models in 2004 with 100% specificity trained on Tomey CASIA 2 imaging [123]; Kundu G. et al. (2023) presented an RF AI model trained on CSO MS-39 imaging, achieving 99.1% accuracy [127]; in 2023, Kundu G. et al. published a Pentacam RF AI model for keratoconus prediction and classification (76% correctly predicted progression and 67% no progression) [125]; Cohen E. et al. published an RF AI model in 2002 that included 94 instrument-derived parameters and device-integrated prediction indices with 91.5% accuracy and 94.7% sensitivity and 89.8% specificity [128]; Tan Z. et al. published a 5-layer feed-forward NN in 2002, reporting 99.6% accuracy and 99.3% sensitivity and 100% specificity [130]; Shetty, R. et al. published a study in 2021 on five AI models reporting 85–89% sensitivity and 82–91% specificity [115]; Xie Y. et al. published a study on a PIRSS CNN in 2020, reporting 94.7% accuracy, 91.9% sensitivity, and 98.7% specificity; [58] and finally, Kamiya, K. et al. (2019) published a study on DL, reporting 0.976–0.993 accuracy [131] (Table 4).

##### Multimodal AI’s Role in Improving Corneal Ectasia and Keratoconus Screening for Refractive Surgery

Multimodal AI is an emerging technology that represents the integration of various device data into a single coherent AI pipeline which uses several different AI models sequentially, each best suited to performing a specific task or processing a specific type of data. Multimodal AI thus integrates different data types into a single coherent AI pipeline. Agentic AI is the autonomous function of AI programs called “agents.” Since there is significant overlap between multimodal AI (different AI models for processing different data types) and Agentic AI capabilities (multiple agents running simultaneously, each processing different data types), “Agentic AI” can be considered a newer, more fully fledged version of multimodal AI technology. Two pilot studies by Wang M. H. in 2025 and by Wang, M. H. & Qin, S. in 2026 are notable for exploring the use of multimodal/agentic AI for keratoconus detection [116,119]. These studies combined LLMs with CNN Visual Image Transformers and other AI backbone technology [116,119]. In this AI pipeline, multiple AI models work sequentially to analyze different data types to establish the correct diagnosis/screening of corneal ectasia [116,119]. Wang M.H.’s 2025 pilot study reported 91% precision and 90% recall using LLM + CNN-ViT [117] and, in the 2026 study, Wang, M. H. & Qin, S. reported an excellent AUC of 0.95 and fast processing time of 95 *±* 12 s per case [116] (Table 4).

Multimodal AI also applies to studies that use different AI processing techniques and dual-device or multiple-device data sources. Dual-device keratoconus studies can thus be considered the first multimodal AI studies in keratoconus. Furthermore, there is significant clinical validation in these studies. To our knowledge, one of the first studies to fit this description was that of Ambrósio R. Jr. et al. in 2017, who introduced the Tomographic and Biomechanical Index Version 1 (TBIv1) [133], an AI tool for detecting ectasia using combined Oculus Pentacam + Oculus Corvis ST (Oculus Optikgeräte GmbH, Wetzlar, Germany) corneal biomechanical data [133]. Oculus Corvis ST is a non-contact tonometer that uses an ultra-high-speed Scheimpflug camera to monitor corneal deformation by calculating several parameters relating to phases of corneal deformation, the deformation amplitude radio between the apex and at 2 mm from the apex, and corneal stiffness at first applanation, among others [133]. The Oculus Corvis ST device produces its own keratoconus index, the Corneal Biomechanical Index (CBI). Ambrósio R. Jr. et al. published a multimodal AI study reported that the TBIv1 index had 100% sensitivity to detect frank ectasia cases (AUC 1.0, KC and VAE-E groups) [133] and excellent AUC of 0.996 for detecting ectasia (KC, VAE-E and VAE-NT groups), correctly classifying 97.5% of cases with 96.2% sensitivity and 98.8% specificity [133]. In total, TBIv1 had statistically higher AUC than all other parameters for every analysis performed, except for the comparison with BAD-D for detecting clinical ectasia cases (KC and VAE-E groups) in which AUC was still higher for TBIv1 at a perfect 1.000 compared to 0.997 for BAD-D [133]. Thus, the Random Forest (RF)-based AI model TBIv1 outperformed the other available software screening tools [133]. In the more recent study by Ambrósio, R. et al. from 2003, the newer RF algorithm TBIv2 [126], developed with 18 features in 156 trees using 10-fold cross-validation, had significantly higher AUC 0.945 for detecting VAE-NT versus the original TBIv1 and a similar 0.999 AUC for detecting clinical ectasia; for all cases, TBIv2’s AUC of 0.985 exceeded TBIv1’s of 0.974 (*p* < 0.0001) [126]. Most recently, Du, K. et al. published a very large study evaluating six ML models using dual-device data (Oculus Pentacam imaging + Corvis ST biomechanical data) in a large multi-cohort setting [120], reporting an AUC of 0.975 for Forme Fruste Keratoconus (FFKC), 0.991 for subclinical keratoconus, and a perfect AUC of 1.00 for keratoconus [120].

##### Performance of AI Models Is Superior Versus Traditional Keratoconus Scoring Systems

The highest performance in automated keratoconus and corneal ectasia screening is currently achieved by AI, using both topographical data from various platforms (Oculus Pentacam, AS-OCT platforms) as well as biomechanical data from the Oculus Corvis ST Device. The exceptional performance of AI models is in stark contrast to traditional keratoconus scores. For example, Mohammadpour M. et al. reported that AI ML classifiers were superior (sensitivity 84.62% and specificity 90%) to the factory-integrated Belin/Ambrósio Enhanced Ectasia Total Deviation Value (BADD) (only 55.56 sensitivity and comparable 86.08% specificity) [129]. Xie Y. et al.’s 2020 study compared the PIRSS AI model to Topographic Keratoconus Classification (TKC), finding that the overall accuracy of TKC, 81%, was inferior to PIRSS at 95%, and that TKC only correctly identified 4 out of 20 (20% accuracy) suspect images [58]. TKC performed similarly to PIRSS for other discrimination categories, with 96.3% accuracy (95% CI 89.5–98.7%) compared to PIRSS’s 98.8% accuracy (95% CI, 93.3–99.9%) [58]. PIRSS also outperformed BAD-D, with a total accuracy of 93.7% (95% CI, 86.2–97.3%) compared to 86.2% (95% CI, 77.0–92.1%) [58]. The performance of the AI models can be further improved by adding more parameters and/or features. Ambrósio, R. et al. compared the AI Tomographic-Biomechanical Index Version 1 (TBIv1) with Version 2 (TBIv2), a novel RF algorithm which was developed with 18 features in 156 trees using 10-fold cross validation [126]. They reported that the AUC of TBIv2 when detecting Very Asymmetric Ectasia (VAE-NT) significantly increased to 0.945, compared to 0.899 for TBIv1 [126]. Both the older TBIv1 and the newer TBIv2 had exceptional performance when detecting clinical ectasia: TBIv1 AUC: 0.999 (98.5% sensitivity and 98.6% specificity, cutoff 0.5); TBIv2 AUC: 0.999 (98.7% sensitivity and 90.1% specificity, cutoff 0.43) [126].

##### Performance of AI Models in Corneal Ectasia Screening Is Similar to Senior Ophthalmologists; AI Can Be Helpful in the Diagnosis of Early or Suspect Keratoconus

In their 2020 study, Xie Y. et al. directly compared the performance of a DL model built on InceptionResNetV2 CNN (PIRSS) versus senior ophthalmologists who perform refractive surgery (“Comparison of Human-Machine with Machine-Machine” table, Xie Y. et al.) [58]. The AI model PIRSS was slightly more accurate (95% CI 88.8–97.8) versus senior ophthalmologists (92.8% accurate, 91.2–94.4% CI). The variance of the results was lower (tighter CI interval) in senior ophthalmologists and greater in the PIRSS AI model [58]. Furthermore, Xie Y. et al. identified the diagnosis of suspect and early KC as a weak point of senior ophthalmologists due to incomplete clinical picture/clinical dilemma, a point where PIRSS also obtained similar sensitivity: for suspect KC, PIRSS achieved 80% versus 83% for senior ophthalmologists; for early KC, PIRSS obtained 95% versus 87% for senior ophthalmologists [58]. Cohen E. et al. published a very large clinical study (8526 tomography images, 4904 eyes) and estimated that the results of the two RF algorithms are similar to that of an experienced corneal expert [128]. The study reported 90.2% accuracy with 94.2% sensitivity and 89.6% specificity for the 94-parameter RF algorithm, and 91.5% accuracy with 94.7% sensitivity and 89.8% specificity for the second RF algorithm with 94-parameters + the tomographer’s KC detection algorithms [128].

##### Eye Rubbing and Corneal Ectasia

Giap B. D. et al. published an interesting study in 2025, which used 6 DL models to interpret data from wrist-based wearable devices [118]. The authors reported that the DL model could highly accurately detect eye-rubbing, with an AUC of 98.26 ± 0.92% and 96.7% on the testing set [118]. The model required 15.32 milliseconds per segment and could potentially be used for real-time operation as a potential tool to support ophthalmologists and researchers [118].

##### Detecting Prior Keratorefractive Surgery

An interesting application of AI technology as it relates to screening patients for refractive surgery is the automated detection of previous laser-assisted refractive surgery. Assaf, J. F. et al. published a very large study in 2025 on 2278 eyes of 1166 patients that sought to distinguish patients with previous keratorefractive laser procedures using AS-OCT and DL AI algorithms [96]. The authors reported a very high accuracy for the DL algorithm of 96%, with 96% F1 score and macro-average F1 score. The DL algorithm effectively differentiated between hyperopic and myopic surgery with 90% accuracy [96]. In another 2025 study, Hong J. et al. used a CNN AI model to process five AS-OCT maps: pachymetry, epithelial thickness, posterior mean curvature, anterior axial power, and anterior stroma reflectance [97]. The CNN model correctly detected myopic LASIK surgery in 90.2 ± 3.6% of cases with 93.5 ± 5.2% sensitivity and 97.8 ± 1.7% [97]. The CNN model also correctly detected hyperopic LASIK with an AUC of 98.2 ± 1.9% and sensitivity of 90.2 ± 5.8% [97].

#### 3.2.3. Predicting Visual Results of the Surgery

Achiron A. et al. published a very large single-center ML study in 2017 on 17,592 PRK or LASIK cases which sought to predict the visual results of the surgery [104]. The authors used a decision forest algorithm, which is an ensemble of decision trees, in order to predict results and understand the risk of unsuccessful outcome based on preoperative patient demographics and clinical data [104]. Prediction of efficacy was based on the use of multiple regression trees into a regression forest—which combines the predictions of several regression trees into a final prediction. The regression forest was built in a stage-wise fashion via a Gradient Boosted Machine algorithm (Extreme Gradient Boosting, XGBoost) [104]. To validate predictive performance of the trained method, the standard 10-fold cross-validation procedure was repeated five times [104]. Patients were grouped into “low efficacy” and “high efficacy” groups [104]. Achiron A. et al. reported that the efficacy predicted by the AI model correlated well with the actual post-operative efficacy (r = 0.72, *p* < 0.001) [104]. The statistical classifier algorithm identified preoperative subjective Corrected Distance Visual Acuity (CDVA) as the top feature [104]. The AI model had an AUC (ideal = 1) of 0.943 for the 0.3 efficacy threshold, 0.913 for 0.5, and 0.887 for 0.8, with specificity at 0.913, 0.894, and 0.845, respectively, and sensitivity of 0.951 at an efficacy threshold of 0.3, 0.935 for 0.5, and 0.904 for 0.8 [104].

In 2023, Wan Q. et al. study used video frames from the Zeiss VisuMax laser (Carl Zeiss Meditec, Jena, Germany) and a combination of DL Resnet50 + 7 ML algorithms, including an XGBoost model, to predict postoperative visual acuity after SMILE [77]. They reported a high prediction accuracy of 96%, and an AUC of 0.962 in training and 0.998 in validation data sets [77]. For the test dataset, the AUC of the XGBoost model was 0.889 (95% CI 0.667–0.889) [77]. The authors used Gradient-weighted Class Activation Mapping (Grad-CAM) to calculate the key regions predicted by the model on SMILE scanning images and visualize the heatmap of the model’s final convolutional layer [77].

Zhai J. et al. published a study in 2026 on AI in SMILE using ML nomograms with Python version 3.8.5. (Python Software Foundation, Wilmington, DE, USA) to distinguish between patients with good (Uncorrected Distance Visual Acuity (UDVA) ≤ 0.1 logMAR) or poor recovery (UDVA > 0.2) at 24 h postoperation [75]. Four ML algorithms were used: linear regression, decision trees, Random Forests, and Neural Networks, with hyperparameters optimized using grid search and 5-fold cross-validation on the training set [75].

Perez E. et al. published an ML study on 120 eyes of 102 keratoconus patients [113], where the predictable accuracy of AI models was obtained in ring segment implantation with either symmetric (FERRARA RING, AJL Ophthalmic, Vitoria-Gasteiz, Spain) or asymmetric segments (AJL PRO+, AJL Ophthalmic, Vitoria-Gasteiz, Spain; INTRASEG, Dunstable, United Kingdom and Emetrop’, Le Boulou, France) with an arc of 150° or 160° and thickness between 150 and 300 microns (µm) [113]. Topography data from MS-39 (CSO, Firenze, Italy) were imported as .CSV files into a series of AI models: a binary classification model via Gradient-Boosting Classifiers (XGBoost) to predict the visual gain of 1 visual acuity line postoperatively, one primary regression model (CatBoost) to predict logMAR values, keratometry, and asphericity, and CNN to process spatial information from the MS-39 topography maps [113]. The CNN model consisted of multiple convolutional layers, max-pooling, and fully connected layers [113]. The authors reported that the CNN models (RMSE of 0.11, R^2^ of 0.10) demonstrated fewer promising results versus the traditional ML models. The CNN architecture was particularly effective at capturing spatial patterns in the corneal maps that are not evident in scalar features used by traditional ML models. CatBoost demonstrated superior accuracy for all targets such as predicting keratometry (R^2^ = 0.76) and visual acuity (R^2^ = 0.59, Line Mean Absolute Error (MAE) = 0.7 lines) [113]. The authors concluded that the ML models could predict which patients would benefit from ICRS implantation, with the Gradient Boosting Classifier correctly predicting which patients would gain more than 1 line of visual acuity postoperatively (R^2^ = 1.0) [113]. Furthermore, the model identified that patients with poorer visual acuity and steeper corneas, or who received asymmetric rings, were more likely to improve vision after surgery [113]. Interestingly, the authors’ analysis showed that there was minimal contribution from the parameters related to the ICRS (dimension, placing); instead, corneal measurements (keratometry, K1, K2, Kmax, corneal asphericity) and preoperative logMAR visual acuity were more determinant of postoperative results [113].

#### 3.2.4. Detecting Intraoperative Complications in SMILE

Wan Q. et al.’s 2023 AI model also identified intraoperative complications, focusing on the edge of the femtosecond laser for the opaque bubble layer (OBL) or the central area for black spots (BS) [77]. The AI model correctly distinguished Loss of Suction (LS) in 100% of cases, OBL in 96%, vs. in 85%, and correctly identified 97% of normal-going cases [77]. The authors concluded that a DL model could help identify intraoperative complications [77].

##### Investigating Coupling Effect in SMILE

Darzi S. et al.’s 2024 study used ML to analyze the magnitude of the coupling effect in two keratorefractive lenticule extraction (KLEx) systems: Smartsight (ATOS; SCHWIND eye-tech-solutions GmbH, Kleinostheim, Germany) and SMILE VisuMax 500 (Carl Zeiss Meditec, Jena, Germany) [95]. The authors used simple linear regression to evaluate the impact of the treatment plan on its own pre–post changes and deviations, and multiple regression (ridge regression to tackle multicollinearity from strong correlations and lasso regression) [95]. The study reported a coupling effect of 8% of cylinder into the sphere and 2% coupling of sphere into cylinder for the VisuMax 500, and for high astigmatism the sphere had a 16% impact on the cylinder [95]. For the SCHWING ATOS, the study reported a 1% coupling effect of sphere into cylinder [95]. Astigmatism was undercorrected by 6% in the VisuMax 500 system and by 4% in the ATOS system, with a bias of 0.05 D; cardinal astigmatism was undercorrected by 9% with a −0.02 D bias in VisuMax 500 and by 1% in ATOS [95]. The authors reported that the ML findings are relevant for cylinder and cardinal astigmatism (R^2^ close to 0.1, low signal-to-noise ratio), contributing to the reduction in residual error [95].

#### 3.2.5. Predicting Refractive Results of the Surgery and Fine Tuning Laser Ablation Parameters. Nomogram Models for SMILE

Zhai J. et al. published a large retrospective study in 2026 on 1253 eyes (632 patients) using an ML-derived nomogram to predict the refractive outcome of SMILE. As previously mentioned, the authors used four ML algorithms: linear regression, decision trees, Random Forests, and Neural Networks. Their hyperparameters were optimized using grid search and 5-fold cross-validation on the training set [75]. The RF model exhibited the best performance, with 95% accuracy, 96% sensitivity, and 94% specificity [75]. For detecting suboptimal refractive outcomes, the RF model exhibited 0.915 AUC, 91.2% accuracy, 83.1% sensitivity, and 92.7% specificity [75]. Overall, the model was good at estimating Manifest Refraction Spherical Equivalent (MRSE), with predicted average MRSE of −0.08 ± 0.32 D, with 92.3% of eyes falling within ±0.50 D and 99.4% within ±1.00 D [75].

Liu P. et al.’s 2025 study on 1025 eyes compared manual target Diopter correction (such as overcorrecting by 10% via “experience” by a junior-resident) to a six-algorithm ML nomogram combining XGBoost, Gradient Boosting Regression (GBR), RF, LightGBM, and Linear Regression (LR) [76]. The ML-derived nomograms averaged −0.92 D versus the actual nomogram using postoperative data −0.93 ± 0.20 D; the SVR ML nomogram averaged −0.91 ± 0.19 D [76]. Overall, all ML models performed exceptionally well compared with the actual postoperative nomogram, with the XGBoost AI model exhibiting the best fitting precision (R^2^ = 0.859, mean value of −0.92 ± 0.19 D) [76]. Root Mean Absolute Error for the six AI models ranged from 0.075 to 0.110, and Mean Absolute Error (MAE) between 0.055 and 0.085 [76].

Luft N. et al.’s 2023 study on SMILE nomograms used a variety of AI models, including a linear model (LM), a Generalized Additive Mixed Model (GAMM), and an NN [78]. They reported RMSEs of 0.355 for LM, 0.348 for GAMM, and 0.367 for the NN, and for astigmatism, 0.279 for the LM, 0.278 for the GAMM, and 0.290 for the NN [78]. The created models resulted in an improvement of eyes within ±0.50 D from 82 to 83% for the LM and NN models, and from 82% to 84% for the GAMM [78]. For astigmatism within ±0.50 D, this improvement would be from 90% to 93% for the LM and GAMMs and from 90% to 92% for the NN model [78].

Park S. et al.’s large 2021 study on 3034 eyes used several ML models to construct a SMILE nomogram for spherical and cylindrical diopter values and for the astigmatism axis: these were multiple linear regression, decision tree, AdaBoost, XGBoost and multi-layer perceptron [79]. The AdaBoost model achieved the highest performance, with RMSEs of 0.1378 for sphere, 0.1166 for cylinder, and 5.17° for the astigmatism axis, respectively [79]. They reported accuracies with a 0.25 D and 25° axis threshold at 0.696 for sphere, 0.976 for cylinder, and 0.994 for cylinder axis [79]. The authors concluded that simpler models, such as AdaBoost or the decision tree, are highly effective and, thus, using NN or high-end models might not always be necessary [79].

Cui T. el. al.’s 2020 comparative study found no statistically significant difference between a surgeon-set SMILE nomogram and a ML model trained on 865 ideal cases [81]. However, the authors reported a higher efficacy index for the ML group (1.48 ± 1.089) versus the surgeon group (1.3 ± 0.27), with 83% of surgeon nomograms within ±0.50 D versus 93% of ML nomograms within ±0.50 D [81].

#### 3.2.6. Assessing Corneal Impact of Laser-Assisted Refractive Surgery

Machado A. P. et al. presented a pilot study to develop a predictive computational model that objectively represents the impact of laser-assisted refractive surgery on corneal tissue [60]. The Relational Tissue Altered (RTA) NN incorporated corneal pachymetry, flap thickness, ablation depth, and patient’s age [60]. RTA min had the highest AUC performance at 0.913, followed by RTA apex at 0.908 [60], followed by the traditional Residual Stromal Bed (RSB min = 0.832, apex = 0.825) and Percent Tissue Altered (PTA min = 0.805, apex 0.800) [60]. Overall, RTA achieved 76% sensitivity and 89.2% specificity, while PTA achieved 66% sensitivity and 85.4% specificity, and RSB min only 52% sensitivity but 91.8% specificity [60]. The authors defined an RTA value above 0.24 as indicative of significant biomechanical impact from the refractive procedure [60]; at values over 0.24, the likelihood of inducing postoperative ectasia was increased. The authors further recommended combining a tomographic/biomechanical index such as TBIv2 with the RTA, such that if the TBIv2 is borderline for a patient, then the presence of RTA above 0.24 should prompt consideration of alternative procedures such as PRK or phakic IOLs [60]. The study recommended further research on integrating RTA with other indices for synergistic potential [60].

#### 3.2.7. Predicting Risk of Corneal Ectasia After Laser-Assisted Refractive Surgery

Santhiago M.R. et al. used an AI model to identify cases at high risk for corneal ectasia after performing LASIK surgery [98]. The comparative case–control study included 339 eyes with normal preoperative topography, of which 65 eyes developed corneal ectasia after LASIK (ectasia group) and 274 eyes did not (control group) [98]. A ML algorithm was developed to analyze 6 features (Percent Tissue Altered (PTA), Residual Stromal Bed (RSB), corneal thickness, flap thickness, central ablation depth, and age) [98] and construct 14 additional features by computationally creating, transforming, extracting, and selecting variables using the ML algorithm [98]. PTA was defined as (flap thickness + ablation depth)/preoperative central corneal thickness [98]. After iterative testing, the final AI model used two of the original variables (PTA and corneal thickness) and two derived via feature engineering process (derivative PTA and Age-Weighted Value (AWV)) [98]. The AI model was tasked with identifying cases at risk for corneal ectasia, and the results were compared to the actual cases that developed corneal ectasia [98]. The authors used a SHAP summary plot to show values for all features and instances, thus revealing the importance of each feature for the AI model [98]. The model placed the most importance on the PTA derivative, followed by PTA, AWV, and corneal thickness [98]. The AI model achieved a near-ideal AUC of 0.99, recall of 0.98, and precision of 0.77 [98]. The ML model correctly predicted all but one case that developed post-LASIK corneal ectasia [98]. The measured classification accuracy for the AI model was 99% versus only 87% from PTA and a meager 22% from the Ectasia Risk Score System (ERSS) [98]. Furthermore, the authors reported that neither RSB nor LASIK flap thickness played a relevant role in separating patients in the ML process [98].

A study by El-Naggar, M. T. et al. of 24 eyes from 15 patients that developed post-LASIK corneal ectasia highlights the power of AI models in potentially detecting patients at risk for post-LASIK corneal ectasia [99]. In this study, the ERSS was suggestive in 62.5% of cases and BAD in just 50% of cases, while the AI-based Pentacam Random Forest Index (PRFI) was suggestive in 87.5% of cases [99].

Another interesting development for assessing surgical risk is the software AcuSimX (Narayana Ophthalmic Research and Development LLP) developed by Francis M. et al. [100], which uses an Inverse Finite Element Model (iFEM) + Surgery-specific Finite Element Model (sFEM) for the human cornea and Oculus Corvis-ST and Oculus Pentacam measurements to simulate the impact of PRK, LASIK, or SMILE surgery [100]. For the SMILE technique, Francis M. et al. analyzed 10 eyes with post-SMILE ectasia using AcuSimX (which combines iFEM, sFEM, and an AI model) [100]. The authors explored discriminative statistical analysis using a decision tree classifier and reported that ectasia eyes had an AcuSimX postoperative Kc_mean_ (Postoperative corneal stiffness, Newton (N)/meter (m)) ≤ 82.85 N/m [100], an Oculus Corvis ST preoperative applanation 2 times less than or equal to 21.67 milliseconds, and an optical diameter greater than 6.5 millimeters (mm) (if preoperative Maximum Deformation Amplitude DA_max_ ≤ 1.187 mm) or a spherical power correction greater than 6.5 Diopter (D) (if preoperative DA_max_ > 1.187 mm) [100]. The authors used a decision tree classifier to better understand and improve the results calculated by AcuSimX, with the AI classifier exhibiting a high AUC of 1.00, precision of 0.99, and recall of 0.99, for a threshold cutoff of 0.92 [100]. The authors reported significant processing time of 1.47 h for the AcuSimX simulation on a medium-spec system (8 core 14 nm-generation CPU and 8 GB of RAM) [100]. Overall, the AI model + AcuSimX combination is helpful for research purposes [100], with a need for further AI optimization for faster processing times and better device integration with refractive platforms currently in practice.

#### 3.2.8. Surgical Planning Assistant—Still in the Works

Surico P.L. et al. studied the performance of OpenAI Chat GPT 5 in refractive surgery planning for myopia or myopic astigmatism on the ELITA-SILK platform (Johnson & Johnson Surgical Vision, Inc., Milpitas CA, USA) compared with expert refractive surgeons [73]. For the spherical component, there was a statistical difference (*p* = 0.0025) with the AI correction being more conservative (−4.29 D ± 2.42 D) than the expert surgeons (−4.70 ± 1.98), with a mean bias of −0.41 in favor of GPT-5 suggestions [73]. For the cylindrical component, no statistically significant difference was found: −0.91 ± 0.69 D for GPT-5 versus −0.92 ± 0.80 for expert surgeons [73]. Importantly, GPT-5 did suggest very different axis orientations for cylinder correction compared with expert surgeons, with the mean axis difference being 61.9° ± 50.1°, and only 9% of GPT eyes had an axis delta within 5°, 12.8% within 10°, and 18% within 15° [73]. The authors showed that GPT-5 based-correction was inferior to established ML nomograms for SMILE; a better-use case of AI [73]. Furthermore, differences in spherical component planning and the high difference in cylinder axis were important limitations of the GPT-5 model, most probably due to GPT-5 not being trained on ophthalmic datasets [73]. This study exemplifies that AI performance is strongest when the training data are highly specialized for the application, and using an off-the-shelf LLM might be a suboptimal approach.

Garnier S. et al. published a proof-of-concept ML study using a retrospective 2448 completed cases treatment database using the excimer laser TENEO 317, Model 1 (Technolas Perfect Vision GmbH, Bausch + Lomb, München, Germany) and FEMTO LDV Z2 (Ziemer Ophthalmic Systems AG, Brügg, Switzerland) [71]. The AI model used 254 objective features from preoperative, postoperative, and intraoperative variables, which included topography data from ORBSCAN 3 (Technolas Perfect Vision GmbH, Bausch + Lomb, München, Germany) and aberrometry data from a Shack–Hartmann wavefront-sensor aberrometer ZYWAVE 3 (Technolas Perfect Vision GmbH, Bausch + Lomb, München, Germany) [71]. The model used several ML techniques, such as LINear Regression (LINR), Bagging Aggregator Gradient Boosted (BAGB), Vanilla Multilayer Perceptron (VMLP), and Bayesian Dynamic MultiHead Neural Network (BDMH) [71]. The LINR predicted defocus accurately (R^2^ = 0.98) but underperformed for Q-factor (R^2^ = 0.47) [71]. The non-linear models achieved better fits for the Q-factor target—BAGB R^2^ = 0.66, VMLP R^2^ = 0.65 and BDMH R^2^ = 0.71—while also offering the excellent predicted defocus accuracy R^2^ = 0.98 [71]. For astigmatism on the Y axis, all models achieved R^2^ = 0.98, for astigmatism on the X axis, values varied more with BAGB and VMLP, achieving a smaller R^2^ = 0.77; LINR fared slightly better with R^2^ = 0.79; and BDMH performed better, R^2^ = 0.87 [71]. The authors noted that the Q-factor varies nonlinearly and depends on interactions among optical zone diameter and preoperative optics [71]; as such the achieved Q-factor accuracy is a noteworthy result on which to carry out further research. Another strength of the ML study is the multi-output prediction: since changing defocus induces or attenuates asphericity, tuning Q-facto shifts defocus, and all these affect the optical zone [71], the ML models can offer direct multi-output prediction without the need for parameter guesswork.

#### 3.2.9. Current-Generation and Next-Generation AI Ray-Tracing Ablation Profiles

The precision and outcome of refractive surgery can be significantly increased by understanding and measuring the eyes as a complete optical system and optimizing the corneal ablation profile so that the optical system becomes nearly devoid of any optical aberration. Personalized ablation profiles thus seek to remove both Lower-Order Aberrations (sphere, cylinder) as well as removing as much as possible from Higher-Order Aberrations (HOAs). Understanding and measuring the HOA layer preoperatively is important, since the optical system of the eye can compensate a certain amount of HOAs and, as such, removal of the HOA removes the optical system from the optical compromise state, leading to significant—possibly supraphysiological—postoperative vision improvement. To our knowledge, Stulting R.D. et al.’s 2016 study on topography-guided LASIK [175] and Kanellopoulos A. J.’s 2016 study of Topography-Modified Refraction (TMR) in myopic LASIK [16] marked the beginning of the current era of topographically customized treatment. This study reported superior visual (1 line of vision gained 55.6% TMR versus 27.8% standard, 2 lines of vision gained 11.1% TMR versus 5.6%) and refractive results (over −0.50 D from target, only 11.7% in TMR versus 27.8% in standard group) [16]. To our knowledge, one of the first customized topographical treatment approved for patient use with FS-LASIK was Alcon^®^ Wavelight^®^ Contoura^®^ (Alcon Laboratories, Inc., Fort Worth, TX, USA), as presented in the Layer Tolked Reduction of Astigmatism (LYRA) Protocol presented in 2017 by Motwani M. in a series of three studies [17,18,19]. LYRA sought to remove the HOA pattern entirely, resulting in a more uniform cornea [19]. Motwani M. reported elimination of astigmatism from all but two of the 50 eyes, with one eye having regression with astigmatism [19]. They reported that 80.85% of patients achieved supraphysiological 20/15 vision and 100% achieved ideal 20/20 vision [19].

Customized topographical ablation profiles have the potential downside of decompensating latent lenticular astigmatism or leaving a small residual spherical component uncorrected. Thus, the potential of topographical ablation was for a time limited by the understanding of the patient’s optical system. To better solve these problems in Contoura^®^ topographical ablation, Lobanoff M. et al. proposed the Phorcides Analytical Engine [22,176], which analyzes the optical system and potentially recommends a more suitable corrective target. Lobanoff M. et al. reported good initial results with mean postoperative cylinder of 0.16, lower than the 0.19 D [22] reported by Moshirfar in a comparative study of two topographical (Alcon^®^ Contoura^®^ and Nidek Final Fit™) and one wavelight-guided (Visx iDesign WaveScan Studio™ System) platform [177]. Furthermore, the visual acuity results from Lobanoff M. et al.’s 2020 study were slightly above those of the FDA study for topographical ablation by Stulting R.D. et al. [22], and were better than those of Kanellopoulos [16]. A newer 2022 study by Stulting R.D. and Lobanoff M. et al. at 3 months postoperation reported high UDVA of 20/20 in 100% of Phorcides eyes, 20/15 UDVA in 89% of eyes, and 20/12.5 UDVA in 28% of eyes [22]. Research on the Phorcides Analytical Engine and the published results have been critically reviewed by other authors, such as Wallerstein, A. & Gauvin, M. [178] and Motwani M. [179]. Overall, the Phorcides Analytical Engine highlighted the potential visual improvement gains of adopting more complex calculation formulas for the topographical ablation profile and the complexity of calculating the optical system.

Ray-tracing is a technology with applications in 3D rendering and gaming GPUs (nVIDIA RTX, nVIDIA Corporation, Santa Clara, CA, USA) that is highly effective at simulating optical performance and refraction of both central and peripheral light sources by calculating hundreds or thousands of individual light sources. Ray-tracing is very computationally intensive, yet is the best simulation of real light refraction and reflection. In refractive surgery, ray-tracing can accurately simulate both pre-ablation and the post-ablation optical system, leading in theory to the creation of the most accurate ablation profile; the AI algorithm can simulate potentially thousands of different light refractions from optical center to periphery. Thus, instead of measuring corneal aberrometry by programing a more uniform cornea devoid of HOA and hoping for the best, ray tracing can trace thousands of light rays and simulate both preoperative and postoperative optical performance. Schumacher S. et al. studied the applications of ray-tracing for LASIK, reporting 83.8% of eyes achieving a UDVA of 20/20 or better [83]. In 2020, Kanellopoulos A. J. first published results using the Innoveyes Sightmap Ray-tracing Software (currently of Alcon Laboratories, Inc., Fort Worth, TX, USA) [84], which calculates ray-tracing based on axial length and wavefront data on corneal surfaces, vitreous cavity, and lens [84]. The InnovEyes Ray-tracing Software with AI processing then creates a personalized ray-tracing model for the individual eye for programming the ablation instead of calculating based on a Gullstrand standard eye model [84].

Kanellopoulos A. J. reported an excellent mean refractive error of −0.11 ± 0.09 D postoperatively and mean −0.15 D ± 0.04 D of astigmatism in a 2020 pilot study [84]. In 2024, Kanellopoulos A.J. et al. performed a FS-LASIK nonrandomized multicenter study on 225 eyes (113 patients) with myopia of less than −9.0 D and astigmatism of less than −4.0 D. They reported 20/20 vision or better in 98.1% of eyes, with MRSE within ±0.5 D in 92% of eyes [86]. He, G., & Bala, C. performed a large study on 400 eyes (200 patients) using the InnovEyes ray-tracing algorithm, reporting UDVA above 20/12 in 51% eyes and of 20/10 in 8% of eyes [85]. They reported no statistically significant increase in HOAs after ray-tracing ablation and a reduction in spherical aberration [85]. Cao X. et al. published two studies in 2024 on the InnovEyes Platform [87,88]. The first study compared ZZ InnovEyes ray-tracing formula with the default ablation profile and reported significant advantages in MRSE for ZZ InnovEyes 0.13 D ± 0.30 D versus 0.62 ± 0.40 D for automated, *p* < 0.001 [87]. ZZ InnovEyes achieved UDVA of 20/16 or better in 97.22% of eyes compared to 85.71% in the default automated group at 3 months follow-up, and even achieved a UDVA of 20/12.5 in 50% of eyes versus only 28.57% in the automated group [87]. The second study compared the InnovEyes Sightmap platform with LASIK to topography-guided LASIK, reporting better UDVA for the ray-tracing group with 48% of eyes achieving a UDVA of 20/12.5 or better [88]. Interestingly, ray-tracing LASIK induced a small increase in HOAs and vertical coma aberration and reduced the spherical component; topography-guided LASIK did not alter HOAs that significantly, instead only increasing the vertical coma [88]. The ray-tracing model was more accurate at 3 months postoperation for residual spherical aberration of −0.021 ± 0.031 versus 0.054 ± 0.122 [88].

In 2025 and 2026, further studies were published on InnovEyes Ray-Tracing. Sitto M. et al. compared InnovEyes Ray-Tracing with Topography-guided Custom Ablation Treatment (T-CAT) and reported better UDVA for ray-tracing 20/16 in 70.2% of eyes compared to 64.8% with T-CAT [89]. However, maximal UDVA was higher for T-CAT, with 15.7% of eyes reaching 20/10 UDVA compared with only 2.5% of eyes in the ray-tracing group [89]. At 3 months postoperation, ray-tracing LASIK showed higher mesopic contrast sensitivity under glare and reduced total spherical component [89]. Huang Y. et al. reported comparable efficacy and visual quality using ray-tracing FS-LASIK for both low and high myopia, with excellent results at 6 months postoperation [90]. Wang Y. et al. reported achieving a UDVA of 20/16 in 33% of eyes, compared to 16% in manifest refraction programming [91]. Tan S. et al. reported excellent vectorial astigmatism results using the ray-tracing platform, with a mean difference vector value of only 0.04 D @ 81°, an arithmetic Corrective Index (CI) of 1.08, and a geometric CI of 1.05, close to the ideal of 1.00 [92]. Thananjeyan, A. L., & Bala, C. researched the data acquisition process (scan times, scan quality) and reported the existence of an operator learning curve due to accommodation during wavefront measurement [93]. Yang L. et al. reported very high patient satisfaction in a cohort with high myopia and myopic astigmatism, with 100% reporting clear distance vision and 98.04% satisfied with the surgery [94]. UDVA was 20/20 in 100% of eyes, at least 20/16 in 69% of eyes, and at least 20/12.5 in 16% of eyes [94].

#### 3.2.10. Using AI as an Educational Tool in Refractive Surgery

One of the most common uses in the day-to-day life of LLMs is as a personal teaching assistant for searching, categorizing, and presenting information in an objective manner. As such, whether LLMs can adequately handle scientific medical information about laser-assisted refractive surgery arises as a key question. Upon reviewing the literature findings, we observed that, as LLM technology has progressed, the reported results improved: LLM studies from 2024 reported lower successful answer rates than those from 2025, and (current at the time of writing) those from 2026 show further improvements over the previous year.

##### AI as an Educational Tool for Patients

In 2024, Sensoy E. & Citirik M. questioned several popular LLMs on their understanding of lens, cataract and refractive surgery. This study reported that OpenAI ChatGPT 3.5. only answered 50% of questions, Microsoft Bing 61.4%, and Google Bard 71.4% [111]. Aydın F.O. et al.’s 2024 study with 40 Frequently Asked Questions (FAQs) from patients used two versions of OpenAI ChatGPT: ChatGPT 3.5 correctly answered only 45% of questions, while ChatGPT 4.0. answered 52.5% [109]. Microsoft Copilot was intermediate at 60%, and Google Gemini significantly outperformed other LLMs at 87.5% correct answers [109]. Demir S.’s 2025 study also evaluated the quality of the provided responses in terms of being easy to read and understand [108]. This study found that OpenAI ChatGPT 4.0. performed better in terms of scores versus Microsoft Copilot and Google Gemini, but produced less educative (easy-to-read) answers [108].

In a more recent (2026) study, Zhou M. et al. used OpenAI GPT4, Google Gemini 2.5 Pro, X Grok-3, and DeepSeek R1 to generate literature reviews on nine topics related to SMILE [105]. The results were not entirely satisfactory, with the models having diminished performance on open topics and exhibiting reference fabrication (“hallucinations”) [105].

Petroff, D.J. et al. (2024) is unique in evaluating the accuracy of AI in generating educational illustrations for patients about popular refractive surgery procedures such as LASIK, PRK, and SMILE [112]. The study used DALL-E 3 and MiM to generate the illustrations [112]. The resultant images represented ocular anatomy with inadequate accuracy, presented other inaccuracies, and had lower education value than images produced by human experts [112]. Overall, the study cautioned that AI image generation focused more on presenting vibrant illustrations than on anatomical accuracy or educational content [112].

##### AI as an Educational Tool for Ophthalmology Resident Physicians, Fellows in Refractive Surgery or Medical Students Interested in Ophthalmology

In 2025, Wallerstein A. et al. used a randomized questionnaire with 228 text-only questions from the American Association of Ophthalmology (AAO) Basic and Clinical Science Course (BCSC) on refractive surgery [106]. Wallerstein A. et al. reported that OpenAI O1 achieved a 91.2% score, while ChatGPT 4o achieved 86.4% [106]. Most interestingly, the cohort of ophthalmology residents only achieved a 77% score [106].

In 2025, Attal L. et al. evaluated seven LLMs using 134 specialty questions from the national ophthalmology certification exams and reported that OpenAI’s ChatGPT O1 model achieved the highest overall accuracy of 83.5%, excelling in complex optical calculations (84.1%) and optics questions (82.4%) [107]. ChatGPT O1 demonstrated near-comparable accuracy for both calculated and non-calculated questions (84.1% versus 83.3%) [107]. For refractive surgery, DeepSeek V3 displayed the highest accuracy in refractive surgery-related questions (89.7%), followed by ChatGPT O3 Mini (88.4%) [107].

Tuttle J.J. et al. (2024) sought to evaluate how correctly medical students could gather information on a specialty topic using LLMs as the main search engine, as opposed to manually searching for the information. The study question was defining the Randleman criteria and their five parameters [110]. The students managed to manually find the correct information in 100% of cases, while OpenAI ChatGPT 3.5 only provided the correct definition for 26.1% of students [110], after hallucinating incorrect information such as “Randleman syndrome” or misidentifying the criteria as a corneal ectasia staging system [110].

## 4. Discussion

Definition- and marketing-wise, AI technology is a large umbrella term for a variety of algorithms that process data autonomously within a given set of objectives/commands. The purpose of the AI models in ophthalmology is to analyze clinical data to find patterns, learn image patterns that lead to certain diagnostics, and help ophthalmologists process, sort, and understand large amounts of patient data. AI is an all-encompassing technological envelope with small technological branches that aid various medical specialty domains, including ophthalmology. In recent years, Artificial Intelligence (AI) technology has shown a dramatic leap in capabilities when compared to the original Machine Learning (ML) techniques and pioneering ophthalmology applications such as Maeda N. et al.’s 1995 ML algorithms for keratoconus diagnosis using topographic corneal maps [138] and Chastang P.J. et al.’s ML decision tree classifier algorithm using images from videokeratoscopy published in 2000 [137]. This review paper identifies several important ophthalmological applications. AI models can help in screening and selecting patients for laser-assisted refractive surgery (PRK, LASEK, LASIK, SMILE) [101,102], screen for pre-existent corneal ectasia [55,114,115,116,117,118,119,120,121,122,123,124,125,126,127,128,129,130,131,132,133,134,135,136,137,138], detect prior keratorefractive surgery [96,97], estimate the risk of post-LASIK corneal ectasia [98], predict the visual and refractive results of the surgery [75,77,104,113], assess the corneal tissue impact of laser ablation [60], detect intraoperative complications for SMILE surgery [77], improve surgical accuracy via nomogram programming [75,76,77,78,79,80,81], optimize ray-tracing 3D eye models [85,86,87,88,89,90,91,92,93,94], and provide educational assistance for patients [108,111,112] and novice/resident/fellow ophthalmologists [106,107,110].

### 4.1. Corneal Ectasia, Keratoconus Screening and Diagnosis—The Most Popular AI Application

This review identified corneal ectasia and keratoconus screening and diagnosis as the ophthalmological application of AI with the most published papers and proven clinical benefit of AI. Latest-generation AI models consistently present high sensitivity and specificity for keratoconus diagnosis (for example, the Traumatic and Biomechanical Index TBv1 [133] and TBIv2 [126], and Du. K. et al.’s 2025 study reporting a 0.991 AUC for subclinical keratoconus and a perfect 1.00 AUC for keratoconus [120]). These results must be interpreted with a comparison of internal versus external validation, calibration, and generalizability. Nevertheless, the technology is highly promising. The fulfillment of the revolutionary potential of AI models in keratoconus and corneal ectasia screening is dependent on the factory integration of these novel tools into commercialized devices. AI integration into tomography devices is currently still incipient and far from its potential. To expand, despite keratoconus tools having long had ML/AI options such as that presented in Saad A. & Gatinel D.’s 2010 study on the Nidek Corneal Navigator, only two of the reviewed studies reported using in-device AI models: Mohammadpour M. et al., who used Oculus Sirius Phoenix [129], and El-Naggar, M.T. et al., who used the Pentacam Random Forest Index (PRFI) index for post-LASIK corneal ectasia [99]. Indeed, few ophthalmology practices have both the computational resources and high patient volumes (5000–10,000+ images or 1000+ eyes) required to train a high-performing keratoconus AI model from scratch; as such, most studies on the subject are sourced from high-volume large refractive surgery settings. Furthermore, AI model integration raises several issues such as model bias, depending on the training dataset; data protection and regulations; and the requirement for more complex hardware and software for the corneal topographer/tomograph. Integrating both imaging (for example, Oculus Pentacam) and biomechanical analysis (Oculus Corvis ST) is a two-device-plus-added-AI-hardware problem, which raises cost, workflow, and complexity issues. However, the advantages are clear: performance that can exceed that of expert refractive surgeons, AI assistance to refractive surgeons in training (residents or novice surgeons), and improvements in speed and patient workflow. The greatest benefit by far would be increasing the accessibility of an early keratoconus diagnosis for patients because, considering the performance of AI models at present, a simple one-time screening with an AI-model-equipped topography/tomography device would likely result in a correct early diagnosis of keratoconus that could be later verified and treated by a specialty center. We hypothesize that, with time, more AI will be factory-integrated into devices for keratoconus screening and corneal biomechanical analysis. The greatest potential benefit would be in creating a proprietary or open-source software module that could accommodate swapping between AI models, which would allow corneal topo-/tomographers to easily switch between different clinically verified AI models or update to the latest high-performing models. This path would be preferrable to a piecemeal approach where each company integrates their own AI software, as this latter approach would be slower and more fragmented in its overall results. Furthermore, the piecemeal approach would create difficulty in keeping up with bleeding-edge high-performing AI models. As such, a certain degree of clinical standardization for deploying keratoconus AI models could be desirable for improving ophthalmological access to high-performance corneal ectasia screening.

### 4.2. Improving the Laser Ablation Profile Calculation

The second-largest ophthalmology AI application for laser-assisted refractive surgery would be AI-optimized ablation calculation via special nomogram programming (as seen in several SMILE surgery studies) or via optimization of the computation-intensive ray-tracing calculation process, such as that employed by the Innoveyes ray-tracing algorithm [84].

In the most basic application, nomograms may refer to manual corrective parameters inputted by the refractive surgeon over the default laser calculation; for example, if the patient requires SMILE surgery for myopia, the laser calculates that −4.00 D of correction is necessary, and the surgeon inputs −4.25 D, knowing from experience that this particular laser tends to slightly undercorrect. Of course, this process can greatly benefit from big data analysis, in which AI models excel. Furthermore, using AI-modeled nomograms removes a significant amount of “guesswork” for a novice refractive surgeon as compared to an experienced surgeon. The results of AI nomograms in SMILE are an upgrade, with Cui T. et al. reporting that 93% of ML nomograms were within ±0.50 D versus only 83% of surgeon nomograms [81]. Park S. et al. (2021) reported that simpler AI models are sufficient, with an AdaBoost model achieving the highest performance, with RMSEs of 0.1378 for sphere, 0.1166 for cylinder, and 5.17° for astigmatism axis, respectively [79]. Other authors such as Luft N. et al. have reported more limited benefits to implementing AI nomograms, such as an improvement in eyes within ±0.50 D from 82 to 83% for the LM and NN models and from 82% to 84% for the GAMM [78]; for astigmatism within ±0.50 D, this improvement would be from 90% to 93% for the LM and GAMMs and from 90% to 92% for the NN model [78]. In any case, from the reviewed literature, it seems that applying an optimized nomogram results in either a slight benefit or a statistically significant benefit and, as such, this technology should be clearly integrated into refractive surgery platforms. The proposed mechanism for the refractive surgeon should be similar to that of current-generation Swept-Source Optical Coherence Tomography (SS-OCT) biometry devices such as the Alcon Argos^®^ (Alcon Healthcare, Fort Worth, TX, USA): the biometry device can use pre- and postoperative measurements to “personalize” for the refractive surgeon, optimizing its calculation. Such technology can be implemented into refractive surgery platforms; however, this implementation might be offset by rising costs (buying an SS-OCT biometry or tomography-with-axial-length-measurement device from the same manufacturer, acquiring localized server or cloud infrastructure, purchasing the required software or licenses). However, from a patient benefit perspective, a more accurate calculation is highly advantageous, with the only disadvantage being the requirement for an additional measurement, with the rest of the system/integration working seamlessly in the background.

Ray-tracing technology works slightly differently from nomograms: ray-tracing simulates the refractive passage of thousands of light rays, constructing a 3D eye model which then dictates the exact shape of the required laser surface ablation. The current ray-tracing models from the InnovEyes Sightmap Platform (Alcon Laboratories, Inc., Fort Worth, TX, USA) can effectively 3D model the eye before the surgery and simulate the eye after the surgery. This technology almost eliminates the very low amounts of spherical aberrations that can remain postoperative using previous topography-customized techniques while offering comparative postoperative HOA amounts [89,90,91,92]. The AI model’s application resides in optimizing the ray-tracing calculation process because calculating thousands of individual light rays is extremely resource-intensive; as such, while this is not a 100% AI application such as ML-derived nomograms, AI can definitively help in optimizing ray-tracing calculations. The results for this AI-optimized ray-tracing algorithm are impressive, with Yang L. et al. reporting its successful use for high myopia and myopic astigmatism vision of at least 20/16 in 69% of eyes and at least 20/12.5 in 16% of eyes [94]. He, G. & Bala, C. reported a UDVA above 20/12 in 51% of eyes and of 20/10 in 8% of eyes [85]. Cao X. et al. highlighted the strength of the InnovEyes ray-tracing formula in greatly reducing residual spherical aberration: InnovEyes achieved an MRSE of 0.13 D ± 0.30 D versus 0.62 ± 0.40 of the default automated calculation (*p* < 0.001) [87]. According to Cao X. et al., ray-tracing can offer slightly better results versus topography-guided LASIK, and they reported a better UDVA for the ray-tracing group, with 48% of eyes achieving a UDVA of 20/12.5 or better [88]. From a clinical point of view, performing quality topography-guided LASIK can present a higher learning curve (checking the tear film normality, performing 4 to 8 high-quality measurements per eye in accordance with the manufacturer’s recommendations, comparing surface measurements and eliminating improper ones, fine-tuning the spherical Diopter correction by experience [17,18,19]) versus performing at most several ray-tracing measurements, with the learning curve focused more on detecting accommodation during wavefront measurement [93].

### 4.3. Assisting with Patient Screening and Selection for Refractive Surgery

The third ophthalmology AI application identified was in patient screening and selection for laser-assisted refractive surgery. Several studies support the idea that, in a large clinical practice setting, AI can reliably identify patients most likely to require or desire refractive surgery [101,102,103]. Furthermore, AI can function as an assistant, recommending the surgical technique (PRK, LASIK, SMILE) while offering details of why it reasons towards a certain technique (via SHAP technology or saliency maps). Thus, the refractive surgeon can weight information from the AI assistant and agree or disagree on a case-by-case basis. Having an “assistant” on hand could be especially helpful in decision making for more novice refractive surgeons, provided that the AI assistant performs adequately for the task.

Agentic/multimodal AI is still in its incipient stages in ophthalmology. Multimodal AI can help process multiple investigations from different devices and screen for corneal ectasia or pre-existing keratoconus [116,119,120]. Furthermore, AI can even reliably predict the risk of post-LASIK corneal ectasia [98]. A dichotomy thus arises: the power of agentic/multimodal AI frameworks should, in theory, be most useful to a smaller ophthalmological practice that does not have dedicated keratoconus experts on hand at all times or the support staff needed to quickly assemble a complete medical file of a patient through a variety of high-end investigations; however, due to the sheer cost and complexity of running and verifying the optimal functionality of several AI systems in parallel, this technology currently only makes sense in a very large, high-patient-volume hospital or multi-clinic practice. As such, it is probable that agentic/multimodal AI will first be integrated into high-patient-volume practices, where it could help to create a more effective workflow regarding patient selection and help increase corneal ectasia screening accuracy as well as treatment accuracy via nomograms. Afterwards, the technology could then trickle down towards smaller practices, where it could be more beneficial for both the refractive surgeon and patient. Further questions arise on the ethics and legal liability of patient data generated using LLMs, which are known to “hallucinate,” and patient data protection regulations. The role of AI models as assistants to refractive surgeons in niche applications where the AI excels (keratoconus screening) is clear; however, the exact roles of agentic AI in a clinical setting are still not clearly defined with the currently available technology.

### 4.4. Assessing Surgical Risk

Several studies have reported results using AI models to predict postoperative corneal ectasia. Santhiago M.R. et al. reported that the AI model accurately predicted all but one (99% accuracy) of 65 eyes that developed post-LASIK ectasia [98], compared to only 87% for PTA and just 22% for ERSS scoring [98]. These findings are remarkable, given that traditional interpretative methods for topometric/tomographic parameters from Oculus Pentacam were found by El-Naggar, M.T. et al. to be insufficient for detecting a relatively large number of ectasia cases [99]. In this retrospective case review by El-Naggar, M.T. et al., non-AI indices were inferior: ERSS suggested increased ectasia risk in 62.5% of cases, BAD in 50% of cases, Ambrósio’s relational thickness (ART) in 45.83% of cases, and the PTA index in just 16% of cases [99]. Instead, El-Naggar, M.T. et al. reported the highest accuracy using the AI-based Pentacam Random Forest Index (PRFI), which reached more than 0.125 in 87.5% of cases [99]. It is thus clear that AI models offer superior predictive capabilities. In 2023, Francis M. et al. developed and published the software AcuSimX (Narayana Ophthalmic Research and Development LLP), which uses an Inverse Finite Element Model (iFEM) + Surgery-specific Finite Element Model (sFEM) for the human cornea and Oculus Corvis-ST and Oculus Pentacam measurements to simulate the impact of PRK, LASIK or SMILE surgery [100]. The authors explored discriminative statistical analysis using a decision tree classifier and reported that ectasia eyes had an AcuSimX postoperative Kc_mean_ (postoperative corneal stiffness, Newton (N)/meter (m)) ≤ 82.85 N/m [100], an Oculus Corvis ST preoperative applanation 2 times less than or equal to 21.67 milliseconds, and an optical diameter greater than 6.5 millimeters (mm) (if preoperative Maximum Deformation Amplitude DA_max_ ≤ 1.187 mm) or a spherical power correction greater than 6.5 Diopter (D) (if preoperative DA_max_ > 1.187 mm) [100]. The AI classifier exhibited a high AUC of 1.00, precision of 0.99, and recall of 0.99, with a threshold cutoff of 0.92 [100]. We report on this study because the AI model + AcuSimX combination could be helpful for further research purposes and can be applied across a wide variety of surgical techniques.

### 4.5. Other Developing Applications

This study also identified several niche applications of AI models, such as in detecting eye rubbing (a risk factor) in patients followed up for suspected corneal ectasia [118], detecting prior keratorefractive surgery [96,97], detecting intraoperative SMILE complications [77], investigating the coupling effect [95], and creating a tissue index (the Relational Tissue Altered (RTA) index presented by Machado A.P. et al.) to assess the corneal impact of the surgery [60]. As AI models excel at finding any correlations between preoperative and postoperative data, it is likely that similar applications would be suitable for AI use and research.

### 4.6. Future Directions

The Internet of Medical Things (IoMT) refers to the interconnection of diverse medical devices and systems to enhance the accessibility, efficiency, and effectiveness of healthcare delivery [180]. Multi-module Neural Networks have been studied in other specialties for use in assisting and enhancing certain robot-assisted surgeries [180]. For ophthalmological applications, Thirunavukarasu A.J. et al.’s review found several proof-of-concept studies that demonstrate the technological potential of robot-assisted surgery [181]. The Preceyes Surgical System can aid vitreoretinal surgeons in performing very delicate maneuvers such as epiretinal membrane peeling [182]. The da Vinci Surgical System can aid in excising pterygium, the removal of eyelid tumors, and enhancing the precision of certain corneal procedures such as amniotic membrane transplants [181]. In 2018, Gijbels A. et al. reported the first robot-assisted retinal vein cannulation in a case series of 4 patients with Retinal Vein Occlusion (RVO) [183]. Using robotic assistance, the authors reported the successful injection of anticoagulant into the 100 µm retinal vein for a period of 10 min [183].

Modern laser-assisted refractive surgery, in essence, is already significantly automated/robotized through the use of precise excimer and femtolaser devices depending on the selected surgical technique. Femtolaser technology is used to create the flap in flap techniques such as FS-LASIK, to create a lenticule in SMILE, or to create special incisions for placing intracorneal ring segments (ICRSs). Excimer laser technology is used for stromal ablation to correct Diopters such as in the PRK and Trans-PRK techniques, or in the FS-LASIK technique. The most advanced automated/robotized technique is Trans-PRK, in which the laser sequentially performs epithelial and then stromal ablation without the creation of any incisions, lenticule, or flap. Thus, in Trans-PRK, the surgeon minimally “touches” the cornea, as in the surgical step of debriding the ablated stroma or placing a therapeutic contact lens at the end of the surgery. Further or complete robotization would include automations to other non-laser-assisted surgical steps such as opening the LASIK flap, placing back the LASIK flap after stromal excimer laser ablation, or lenticule extraction in SMILE. For Trans-PRK, this would include automating the steps of applying saline solution to the corneal surface, debriding the ablated stroma, the topical application of mytomicin, and contact lens placement. To our knowledge, there is a lack of studies on the complete automation of refractive surgery techniques as described herein. Furthermore, there is a lack of information on the potential benefits; hypothetically, these could include a reduction in the intraoperative time spent for the patient, increased technical reproducibility, and potential safety benefits for flap placement or lenticule extraction. Multimodal AI that performs image recognition in data processing could potentially also be integrated as a decision-assisting or decision-driving system. Thus, this discussion subchapter highlights a lack of literature data on the integration of multimodal AI with the further automation/robotization of refractive surgery. Laser treatment application is already automated to a certain degree (e.g., in programmed laser delivery, eye tracking for centration and cyclotorsion compensation), and AI is (at the time of writing) used to improve the quality of the ablative calculation and the scientific value of the postoperative feedback, such as in analyzing and interpreting refractive and biomechanical data. Ji Y. et al. suggested that AI-driven decision-support systems could accelerate the transition of refractive surgery from standardized protocols to data-driven, individualized management [51]. Further patient-individualized optimizations could be a benefit derived from AI-analyzed preoperative and postoperative data.

## 5. Conclusions

This review paper highlights important clinical applications of AI models, such as high sensitivity and specificity in the diagnosis of corneal ectasia and keratoconus, as well as popular applications and further developments in application. At present, AI models help refractive surgeons with nomogram programming; optimize ray-tracing ablation calculations; help as assistants with patient selection, visual results, and predicting the risk of ectasia; and assist in more niche applications such as analyzing the laser coupling effect, detecting intraoperative complications, detecting eye-rubbing for ectasia suspects, and analyzing the causes of postoperative corneal ectasia. As such, AI has already arrived in laser-assisted refractive surgery, in the form of specialized programs that excel at specific data-intensive tasks. The advent of SHAP and saliency map technology allows the clinician to observe the mechanistic reasoning of the AI model, find parameters responsible for the increased post-LASIK ectasia risk, choose the particular refractive surgery, and conduct further clinical research. The field retains its novelty aspect, with recently published niche studies alongside established clinical applications. At present, the best-performing and novel AI models are in the domain of large clinical settings or multi-hospital settings, due to the requirement of latest-generation diagnostic devices, large volumes of patient data from high-patient-volume clinics, and significant computational expertise and resources. Integrating the best-performing AI models into ophthalmology diagnostic devices such as topography/tomography devices and corneal biomechanical analysis is the next logical step, with the technology thus trickling down towards smaller-volume refractive surgery practices. Traditional AI models have high potential clinical benefit and specific applicability, and do not suffer from “hallucinations” as is the case for LLM technology; instead, they are judged based on their evaluated accuracy, AUC, sensitivity, and specificity. Agentic/multimodal AI is in its incipient framework stages, with large clinical/patient settings potentially benefiting from the combination of several AI models to extract and process data from different ophthalmological devices, condensing information into an easy-to-read patient file with assessments of diagnosis and patient risk. Complexity, the current limitations of LLM technology (“hallucinations”), data privacy, and ethical and legislative factors represent challenges to overcome for widespread acceptance and utilization of the technology.

## Figures and Tables

**Table 1:** Papers analyzed in the literature review.

Review Papers				
Review Papers on General AI in Ophthalmology Containing Information on Laser-Assisted Refractive Surgery				
Ref. Nr.	Year	Study Authors	Study Title	Study Summary
[45]	2026	Gurnani, B., & Kaur, K.	Artificial intelligence in ophthalmology: from innovation to clinical integration	Generalized summary of Artificial Intelligence (AI) applications: ectasia screening, ocular surface analysis, differential diagnosis in infectious keratitis, retinal imaging, optic nerve head analysis, visual field analysis, AI-based biometry and AI-assisted corneal ablation profiles, multimodal imaging [45].
[46]	2026	Zhang, Z. et al.	Evaluating Large Language Models in Ophthalmology: Systematic Review	Found 187 studies using Large Language Models (LLMs), 169 of which evaluated one to three models and 53.5% compared multiple LLMs. The three most-used LLMs were ChatGPT (90%), Gemini (31%), and Microsoft Bing Copilot (17.1%). Descriptive statistics on LLM use in ophthalmic subspecialties and clinical alignment (24.1% of LLMs used clinical data). The authors reported high heterogeneity on LLM use in ophthalmology [46].
[47]	2025	Kazemzadeh K.	Artificial intelligence in ophthalmology: opportunities, challenges, and ethical considerations	Focused on AI keratoconus detection (reported accuracy 98–99.6%), AI IOL calculation formulas, optic nerve, and retinal imagery and optometric practice. Identified key challenges in implementing AI such as dataset bias, validation, regulatory hurdles, ethical issues, and responsibility for AI-related errors [47].
[48]	2025	Cho, Y. et al.	Artificial intelligence and refractive surgeries including laser vision correction and phakic IOL implantation—a narrative review	General review on AI applications in refractive surgery. Clear explanation of data types encountered in refractive surgery and corresponding types of AI use. Tabular data (numerical and categorical variables, arranged in structured formats) is more suited for traditional Machine Learning (ML) algorithms such as linear regression models, Support Vector Machines, Random Forests, and gradient boosting [48]. Image data (from topo/tomography devices and Anterior Segment Optical Coherence Tomography (AS-OCT) is suitably used by Deep Learning Architecture: Convolutional Neural Networks (CNN) and Vision Transformers (ViTs) [48]. Multimodal data combines tabular parameters and text notes (such as from physician or medical records) with image-based findings. Multimodal AI models or frameworks thus integrate data from various sources to better interpret, diagnose, and aid with clinical or surgical decisions [48]. Reports AI applications in patient selection for refractive surgery, surgical method selection, ablation profile optimization, surgical prognosis, and postoperative measurement [48].
[49]	2020	Lee, J.K. et al.	Artificial intelligence in cornea, refractive and cataract surgery	Reports AI improvements in cornea, refractive, and cataract surgery. In keratoconus, AI improves screening, prevention, and follow-up. The review also focuses on development of next-generation three-dimensionality biometry formulas and big data integration [49].
[50]	2019	Ting, D. et al.	Deep learning in ophthalmology: The technical and clinical considerations	Expanded depth explication of Convolutional Neural Network (CNN) architecture, Deep Learning methodology, training, and performance metrics. Applications in Diabetic Retinopathy (DR), Optic Disc Imaging, visual field analysis, and Age-related Macular Degeneration (ARMD) [50].
Review Papers on AI in Refractive Surgery				
[51]	2026	Ji, Y. et al.	Artificial intelligence in refractive surgery: progress, challenges, and future directions	Generalized summary of AI refractive surgery screening: Machine Learning (ML) algorithms for patient screening, recommending surgical methods. ML use for predicting refractive outcomes after SMILE surgery, predicting myopic regression. Applications of AI in ICL surgery [51].
[52]	2025	Zaheer H.A. et al.	Refractive Surgery in 2025: Innovations, Individualization, and Emerging Frontiers for Treating Myopia	Highlights literature limitations regarding the utility of AI in refractive surgery to date. Reports 95% accuracy and results comparable to senior physicians for ectasia risk screening. Reports innovation in Small-Incision Lenticule Extraction (SMILE) ablation planning and performance using Machine Learning (ML) nomograms [52].
[53]	2025	Hoopes, P. C. et al.	Artificial intelligence in refractive surgery	Highlights emerging studies from Asia (China) and the United States of America. Discusses AI applications in biometry, IOL selection and ICL sizing, and surgical decisions [53].
[54]	2024	Rampat, R. et al.	Artificial intelligence applications in cataract and refractive surgeries	Summary of Deep Learning (DL) studies in general ophthalmology. Refractive surgery topics discussed: keratoconus screening, ICL sizing, and calculation [54].
[55]	2021	Rampat, R. et al.	Artificial Intelligence in Cornea, Refractive Surgery, and Cataract: Basic Principles, Clinical Applications, and Future Directions	Detailed presentation of CNN working principles and model training. AI applications in keratitis differential diagnosis, keratoconus, refractive surgery nomogram and IOL calculation, cataract diagnosis, and grading [55].
[56]	2020	Jayadev, C., & Shetty, R.	Artificial intelligence in laser refractive surgery-Potential and promise!	Condensed editorial paper mentioning corneal ectasia screening for FS-LASIK and keratoconus monitoring. Mentions studies on AI in FS-LASIK and SMILE. Telemedicine applications [56].
[57]	2020	Redd, T. K. et al.	Artificial Intelligence for Refractive Surgery Screening: Finding the Balance Between Myopia and Hype-ropia	Invited commentary paper [57]. Details the Pentacam InceptionResNetV2 Screening System (PIRSS), a tomographic-based DL screening tool presented by Xie. Y. et al. in 2020 [57,58].
Review Papers AI in Keratoconus and Refractive Surgery Screening				
[59]	2025	Esporcatte L.P. et al.	The power of predictive artificial intelligence for the detection of ectasia	Summarizes AI applications in management of corneal ectatic diseases and ectasia risk assessment. Reports that AI-based indices outperform traditional counterparts in detecting subclinical ectasia and identifying risk in fellow eyes after monocular surgery [59]. Highlights the Relational Tissue Altered (RTA) metric (Machado A.P. et al.) [60], which outperformed relational stromal bed and Percent Tissue Altered (PTA) metric in assessing surgical risk [59,60].
[61]	2025	Morales, P., & Durán, J. A.	Advances in Intracorneal Ring Segment (ICRS) Implantation for Keratoconus: A Comprehensive Literature Review, Clinical Insights, and Future Prospects	Focused on innovations in Intracorneal Ring Segment (ICRS) implants [61]. Cites study by Fariselli, C. et al. on using an AI Neural Network (NN) versus producer (KeraRing) nomograms [62]. In the cited study, NN produced better results in visual acuity, Spherical Equivalent (SE), and quality of life of keratoconus patients [61,62].
[63]	2024	Moshirfar M. et al.	LASIK versus PRK Based on Increased Risk of Corneal Haze: Assessing Current Decision-Making Capabilities of Six Artificial Intelligence Models in Refractive Surgery	Assessment of decision-making capabilities of six different LLM AI models for a theoretical patient of −3.50, −5.00 and −7.00 D versus a group of six experienced ophthalmologists. Reported complete dissension between expert opinion (choose LASIK) versus AI opinion (choose PRK). Furthermore, it noted that LLM-provided references were fictitious in 55% of cases [63].
[64]	2024	Afifah, A. et al.	Artificial intelligence as diagnostic modality for keratoconus: A systematic review and meta-analysis	Systematic review on AI diagnostic modality for keratoconus [64]. Found 11 clinical studies, seven of which (63.6%) had high risk of bias (Cochrane Quality Assessment of Diagnostic Accuracy Studies QUADAS-2). Reported high sensitivity for NN and CNN in diagnosing keratoconus; less sensitivity from Random Forest (RF) algorithms. Reported that NN and Naïve Bayes (NB) algorithm have highest specificity for keratoconus diagnosis [64].
[65]	2023	Vandevenne, M. M. et al.	Artificial intelligence for detecting keratoconus	Focused on diagnostic accuracy of AI in keratoconus. Reported 98.6% sensitivity and 98.3% specificity for manifest keratoconus diagnosis and 90% sensitivity and 95.5% specificity for subclinical keratoconus. Reported that diagnostic accuracy may be suboptimal and evidence of low to very low certainty due to indirectness and high risk of selection [65].
[66]	2023	Ambrósio, R. et al.	Multimodal diagnostics for keratoconus and ectatic corneal diseases: a paradigm shift	Multimodal imaging review detailing innovations in keratoconus diagnosis. Reports that AI Model interpretation of Tomographic and Biomechanical Index (TBI) improves diagnostic accuracy even in mild keratoconus cases. Recommends further multimodal integration of corneal tomography and biomechanical parameters [66].
[67]	2023	Kundu G. et al.	Role of artificial intelligence in determining factors impacting patients’ refractive surgery decisions	Retrospective analysis on 423 patients using several AI models: multivariable Logistic Regression, decision trees classifier and RF. RF model provided the best output and correctly predicted 93% of cases as undergoing refractive surgery. Reported AUC of 0.945 with sensitivity of 88% and specificity of 92.5% [67].
[68]	2021	Atalay, E. et al.	Advances in the diagnosis and treatment of keratoconus	Focused on technological advances in diagnosis of keratoconus. Brief mention of the effect of AI DL studies on keratoconus and early disease detection [68].
[69]	2019	Lin, S. R. et al.	A Review of Machine Learning Techniques for Keratoconus Detection and Refractive Surgery Screening	Explores the results of ML studies in keratoconus. Reports very good differentiation of normal and keratoconic eyes and a positive trend towards studies with patients/eyes. Critical view that integrating ML algorithms into topography machines/Anterior Segment Optical Coherence Tomography (AS-OCT) is a necessity for clinical impact of the technology [69].
[70]	2019	Lopes B.T. et al.	Artificial Intelligence in Corneal Diagnosis: Where Are we?	Overview of ML techniques in keratoconus and refractive surgery screening. Reports emerging research at the time on Pentacam images and RF AI models outperforming standard Belin/Ambrósio Enhanced Ectasia Display (BAD-D) and emerging research for in vivo corneal morphology. Highlights the need for large image databases for training keratoconus or refractive surgery models, a limitation resolved by more recent studies [70].

Abbreviations: Ref. nr.—reference number; AI—Artificial Intelligence; LLM—Large Language Model; IOL—intraocular lens; ML—Machine Learning; CNN—Convolutional Neural Network; ViT—Vision Transformers; DR—Diabetic Retinopathy; ARMD—Age-related Macular Degeneration; RTA—Relational Tissue Altered; PTA—Percent Tissue Altered; AS-OCT—Anterior Segment Optical Coherence Tomography; SMILE—Small-Incision Lenticule Extraction; ICL—Implantable Collamer Lens; DL—Deep Learning; FS-LASIK—Femtosecond Laser-Assisted In Situ Keratomileusis; PIRSS—Pentacam InceptionResNetV2 Screening System; ICRS—Intracorneal Ring Segment; NN—Neural Network; SE—Spherical Equivalent; RTA—Relational Tissue Altered Metric; PTA—Percent Tissue Altered; QUADAS-2—Cochrane Quality Assessment of Diagnostic Accuracy Studies; RF—Random Forest; NB—Naïve Bayes; TBI—Tomographic and Biomechanical Index; AUC—Area under the Curve; BAD-D—Belin/Ambrósio Enhanced Ectasia Display.

**Table 2:** Original article papers on laser-assisted refractive surgery analyzed in this paper.

Original Research Papers Detailing the Use of AI in Performing Refractive Surgery				
Ref. Nr.	Year	Study Authors	Study Title	Study Summary
[71]	2026	Garnier S.	Machine Learning-Based Prediction of LASIK Console Inputs for Aspheric Planning (Q-factor, Defocus, Astigmatism): A Translational Methods Study	Feasibility study (not clinical). Evaluated performance of nonlinear models versus linear progression. Supervised nonlinear multi-output models achieved lower error and better calibration [71].
[72]	2026	Bustos-Ampudia, C. et al.	Machine Learning Prediction Models for Classifying Myopic Versus Hyperopic Ablation Patterns in Eyes With Previous Refractive Surgery Undergoing Cataract Surgery	Retrospective analysis on 72 eyes of Random Forest (RF), decision tree and Logistic Regression models using corneal asphericity (Q-value), posterior-to-anterior curvature ratio (P/A ratio), sagittal map morphology, spherical aberration (Z^0^_4_), mean keratometry, central corneal thickness, and thinnest pachymetry. RF models achieved highest discriminative performance (Area Under the Curve AUC = 0.933) versus Logistic Regression (AUC = 0.881) and decision tree (AUC = 0.828) [72].
[73]	2026	Surico, P. L. et al.	GPT-5-assisted versus expert surgeon refractive planning in smooth incision lenticule keratomileusis (SILK): comparative analysis and visual outcomes	Assessed performance of GPT-5 in refractive surgery planning on 134 eyes from 67 patients on the ELITA-SILK refractive surgery platform. Reported difference in spherical component (GPT-5 recommended −4.29 ± 2.42 Diopter (D) correction versus −4.70 ± 1.98 D surgeon recommended) and cylinder axis correction. Favored surgeon-defined correction and cautioned that more clinical validation is needed for integrating LLMs [73].
[74]	2026	Xiong, J. et al.	Predicting Small Effective Optical Zone After SMILE via Multimodal Machine Learning Integrating Corneal Topography and Clinical Parameters	Prediction of the postoperative Effective Optical Zone (EOZ) in SMILE using AI Multimodal Machine Learning (MML) via 16 preoperative parameters (age, gender, eye, sphere, cylinder, axis, steep keratometry, flat keratometry, axis of steep keratometry, corneal diameter, central corneal thickness (CCT), Residual Stromal Thickness (RST), and Lenticule Thickness (LT). Anterior Axial Curvature Map and Preoperative Parameter-Based Model (AACM-PP) obtained 0.997 AUC in low or moderate Spherical Equivalent (SE) correction and 0.958 AUC in high SE correction, 0.871 AUC in low cylinder correction, and 0.667 AUC in high cylinder correction [74].
[75]	2026	Zhai, J. et al.	Personalized prediction of post-SMILE refractive outcomes using a machine-learning nomogram	Large retrospective study on 1253 eyes (632 patients) that underwent SMILE surgery. ML-derived nomogram for predicting refractive outcomes following SMILE. AI model achieved 83.10% sensitivity and 92.70% specificity, 91.20% accuracy, and 0.915 AUC. The paper presents detailed data visualization in the form of histogram and density plots. Reported close alignment between predicted and actual outcomes. Recommended RF as the best-performing algorithm and most suitable for clinical integration [75].
[60]	2025	Machado A.P. et al.	Development and Validation of the Relational Tissue Altered (RTA) Index: Applied Artificial Intelligence for the Assessment of Structural Impact from Laser Vision Correction	Retrospective case–control study of 3278 eyes (1690 patients) to develop a predictive computational model to objectively represent impact on corneal tissue of laser-assisted refractive surgery. Relational Tissue Altered (RTA) Index demonstrated sensitivity of 76% and specificity of 89.2%, superior to Percent Tissue Altered (PTA) sensitivity of 66% and specificity of 85.4% [60].
[76]	2025	Liu, P. et al.	Adopting machine learning to predict nomogram for small incision lenticule extraction (SMILE)	Large retrospective study of 1025 eyes which compared junior-resident-designed nomogram versus ML-designed nomogram. ML provided higher accuracy within 0.05–0.25 D. Reported that ML methods may assist refractive surgeons and shorten learning curve of junior residents in SMILE nomogram [76].
[77]	2023	Wan Q. et al.	Prediction of Early Visual Outcome of Small-Incision Lenticule Extraction (SMILE) Based on Deep Learning	Deep Learning (DL) study using 10,176 femtosecond laser scanning images from 318 eyes (159 patients). DL model Resnet50 was trained to predict postoperative visual acuity. Gradient-weighted Class Activation Mapping (Grad-CAM) was performed to interpret the model. Reported that DL achieved a high accuracy of 96% for image prediction and AUC of 0.962 in training and 0.998 in validation datasets. Reported that ML model XGBoost accurately distinguished patients with good or poor recovery after SMILE. Reported AUC for ML model of 0.998 and 0.889 in the retrospective cohort (training and test datasets) and 1.000 and 0.984 in the two prospective cohorts. Reported training a DL model which accurately distinguished suction loss (100%), black spots (85%) and opaque bubble layer (96%) [77].
[78]	2023	Luft, N. et al.	Optimizing Refractive Outcomes of SMILE: Artificial Intelligence versus Conventional State-of-the-Art Nomograms	Large retrospective study of 1342 eyes which compared nomograms created using a linear model (LM), a Generalized Additive Mixed Model (GAMM) and an AI Neural Network (NN). Results were similar for all three methods with SE accuracy at 82–83% for LM, 85% for GAMM and 83% for NN; for astigmatic outcomes, 90–93% for LM, 93% for GAMM and 92% for NN [78].
[79]	2021	Park, S. et al.	Artificial intelligence-based nomogram for small-incision lenticule extraction	Large retrospective study of 3034 eyes comparing several ML methods for nomogram creation (multiple linear regression, decision tree, AdaBoost, XGBoost, multi-layer perceptron). AdaBoost ML algorithm achieved highest performance with Root Mean Square Error (RMSE) of 0.1378 for sphere, 0.1166 for cylinder and 5.17° for astigmatism axis. Results by accuracy threshold of 0.25 D were 0.257 for sphere, 0.717 for cylinder and 0.596 for cylinder axis [79].
[80]	2020	Shetty, R. et al.	Early Corneal and Epithelial Remodeling Differences Identified by OCT Imaging and Artificial Intelligence Between Two Transepithelial PRK Platforms	Comparative, contralateral eye study on Transepithelial Photorefractive Keratectomy (Trans-PRK) which analyzed epithelial healing and remodeling. One eye was operated on using Schwind SmartSurfACE, while the other eye was operated on using Alcon Streamlight. Reported that Streamlight removed a greater amount of epithelium with comparable visual and refractive outcomes [80].
[81]	2020	Cui, T. et al.	Applying Machine Learning Techniques in Nomogram Prediction and Analysis for SMILE Treatment	Comparative study on SMILE surgery between surgeon-designed nomogram and ML model trained on 865 ideal cases. Reported no statistically significant difference in baseline data. Reported higher efficacy index for the ML group (1.48 ± 1.089) versus surgeon group (1.3 ± 0.27). Proportion of 83% of surgeon nomogram eyes and 93% of ML eyes were within ±0.50 D [81].
[82]	2020	Khamar P. et al.	Epithelium Zernike Indices and Artificial Intelligence Can Differentiate Epithelial Remodeling Between Flap and Flapless Refractive Procedures	Decision tree-customized AI classifier used to calculate epithelial Zernike indices from three-dimensional distribution of epithelial thickness. Reported postoperative change in epithelial Zernike indices and differential changes in epithelial thickness distribution based on the type of surgery. Reported higher analysis accuracy for AI model with all surgical groups classified separately [82].
Studies using AI-optimized ray-tracing algorithm for calculating the ablation profile Ray-tracing algorithms are a potential application for improvement via AI. While traditional ray-tracing path-tracing calculations are different from traditional AI techniques such as Machine Learning, AI optimization of the ray-tracing calculation process is increasingly presented as an AI application. We have thus included ray-tracing studies.				
[83]	2011	Schumacher S. et al.	Optical Ray Tracing-guided Laser in Situ Keratomileusis for moderate to high myopic astigmatism	One of the first studies to use ray-tracing iterative path tracing algorithms. Reported good feasibility of the technology and excellent visual results with 83.8% of eyes achieving UDVA of 20/20 or better [83].
[84]	2020	Kanellopoulos A. J.	Initial Outcomes with Customized Myopic LASIK, Guided by Automated Ray Tracing Optimization: A Novel Technique	One of the first studies using the commercial software InnovEyes Ray-tracing, which calculates the ray tracing based on a 3D digital twin model of the eye constructed out of axial length and wavefront data on corneal surfaces, vitreous cavity, and lens. Reported excellent mean refractive error of 0.11 ± 0.09 D postoperatively and mean −0.15 D ± 0.04 D of astigmatism [84].
[85]	2023	He, G., & Bala, C.	Ray-tracing-guided myopic LASIK: real-world clinical outcomes	A large study on 400 eyes (200 patients) using the InnovEyes ray-tracing algorithm, reporting UDVA above 20/12 in 51% eyes and of 20/10 in 8% of eyes [85].
[86]	2024	Kanellopoulos, A. J. et al.	International Multicenter, Myopic and Myopic Astigmatism Femto LASIK, Customized by Automated Ray-Tracing Ablation Profile Calculation: A Postmarket Study	FS-LASIK nonrandomized multicenter study on 225 eyes (113) patients with myopia of less than −9.0 D and astigmatism of less than −4.0 D, which reported 20/20 vision or better in 98.1% of eyes, with MRSE within ±0.5 D in 92% of eyes [86].
[87]	2024	Cao, X. et al.	Optimization of Ray-Tracing-Guided LASIK Outcomes: A Prospective Comparative Study of ZZ InnovEyes Strategy versus Automated Strategy	Compared ZZ InnovEyes ray-tracing formula versus the default ablation profile and reported significant advantages in MRSE for ZZ InnovEyes 0.13 D ± 0.30 D versus 0.62 ± 0.40 D for automated, *p* < 0.001. ZZ InnovEyes achieved UDVA of 20/16 or better in 97.22% of eyes compared to 85.71% in the default automated group at 3 months follow-up and even achieved UDVA of 20/12.5 in 50% of eyes versus only 28.57% of automated group [87].
[88]	2024	Cao, X. et al.	Study of the InnovEyes Sightmap Platform in Comparing Ray-Tracing-Guided LASIK and Topography-Guided LASIK	Compared the InnovEyes Sightmap platform with LASIK to topography-guided LASIK, reporting better UDVA for the ray-tracing group, with 48% of eyes achieving UDVA of 20/12.5 or better. Interestingly, ray-tracing LASIK induced a small increase in HOAs and vertical coma aberration and reduced the spherical component; topography-guided LASIK did not alter HOAs that significantly, instead only increasing vertical coma. The ray-tracing model was more accurate at 3 months postoperation for residual spherical aberration −0.021 ± 0.031 versus 0.054 ± 0.122 [88].
[89]	2024	Sitto, M. M. et al.	Comparison of U.S. FDA Premarket Approval Studies Between Ray Tracing-Guided LASIK with InnovEyes Sightmap Versus Topography-Guided LASIK Using Custom Ablation Treatment (T-CAT)	Compared InnovEyes Ray-Tracing versus Topography-guided Custom Ablation Treatment (T-CAT) and reported better UDVA of 20/16 in 70.2% of eyes for ray-tracing versus 64.8% in T-CAT. Maximal UDVA was however higher for T-CAT, with 15.7% of eyes reaching 20/10 UDVA versus only 2.5% of eyes in the ray-tracing group. At 3 months postoperation, ray-tracing LASIK showed higher mesopic contrast sensitivity under glare and reduced total spherical component [89].
[90]	2025	Huang, Y. et al.	Changes of refraction and visual quality after ray-tracing guided FS-LASIK in Chinese high myopia versus low-to-moderate myopia	Reported comparable efficacy and visual quality using ray-tracing FS-LASIK for both low and high myopia, with excellent results at 6 months postoperation [90].
[91]	2025	Wang Y. et al.	Refractive outcome and visual quality of Ray-Tracing Guided LASIK: a prospective study	Prospective, observational cohort study of 89 eyes (46 patients) undergoing AI-optimized ray-tracing calculation model for LASIK. Reported that 100% of eyes achieved Uncorrected Distance Visual Acuity (UDVA) of at least 20/25, with 76% of the ≤0.75 D group and 81% of >0.75 D reaching 20/16. Furthermore, 33% and 16% of these groups reached a UDVA of 20/12.5. Overall, reported safe and effective results for Ray-Tracing Guided-LASIK and avoidance of induction of HOAs [91].
[92]	2025	Tan, S. et al.	Ray-tracing-guided FS-LASIK Using Mi Optimized Strategies	Reported excellent vectorial astigmatism results using the ray-tracing platform with mean difference vector value of only 0.04 D @ 81° and arithmetic Corrective Index (CI) of 1.08 and geometric CI of 1.05, close to the ideal 1.00 [92].
[93]	2025	Thananjeyan, A. L. et al.	Factors Affecting Ray Trace LASIK Data Acquisition in Clinical Practice: Options for Addressing Accommodation	Researched the data acquisition process (scan times, scan quality) and reported the existence of an operator learning curve, since accommodation during wavefront measurement can occur [93].
[94]	2026	Yang, L. et al.	Ray-Tracing-Guided Femtosecond LASIK: Refractive Outcomes, Visual Quality and Patient Satisfaction	In a high myopia and myopic astigmatism cohort, Yang L. et al. reported very high patient satisfaction, with 100% reporting clear distance vision and 98.04% satisfied with the surgery. UDVA was 20/20 in 100% of eyes, at least 20/16 in 69% of eyes, and at least 20/12.5 in 16% of eyes [94].
Studies using AI to measure coupling effect in keratorefractive lenticule extraction procedures				
[95]	2024	Darzi, S. et al.	Refractive Coupling Effects in Keratorefractive Lenticule Extraction Procedures: A Machine Learning-Assisted Approach	Retrospective multi-refractive-platform study (2841 SMILE eyes on ZEISS VisuMax 500 and 2528 eyes with SmartSight on SCHWIND ATOS). For VisuMax 500, reported a coupling effect of 8% of cylinder into sphere and 2% coupling of sphere into cylinder; for SCHWIND ATOS, there was a 1% coupling effect of sphere into cylinder. In high astigmatism, plans with VisuMax 500 sphere had 16% impact on cylinder. Suggested that torsional eye movements may partly be responsible for coupling data [96].
Studies using AI to screen for preexisting corneal refractive surgery				
[96]	2025	Assaf, J. F. et al.	Automated Detection of Keratorefractive Laser Surgeries on Optical Coherence Tomography Using Deep Learning	Very large DL study on 14,948 eye scans from 2278 eyes of 1166 patients which used AS-OCT for automatic detection of keratorefractive laser surgeries. Reported DL algorithm accuracy at 96% (including 96% F1 score and 96% macro-average F1 score). Further differentiation between hyperopic and myopic surgery (DL algorithm 90% accurate) [96].
[97]	2025	Hong J. et al.	Artificial Intelligence-Driven Detection of LASIK Using Corneal Optical Coherence Tomography Maps	CNN AI model using five AS-OCT maps: pachymetry, epithelial thickness, posterior mean curvature, anterior axial power and anterior stroma reflectance. Reported that the model accurately detected myopic LASIK in 90.2 ± 3.6% of cases with 93.5 ± 5.2% sensitivity and 97.8 ± 1.7% AUC; for hyperopic laser, sensitivity was 90.2 ± 5.8% and AUC 98.2 ± 1.9% [97].
Studies using AI to assess risk of developing post-refractive surgery corneal ectasia				
[98]	2022	Santhiago, M. R. et al.	Ectasia Risk Model: A Novel Method Without Cut-off Point Based on Artificial Intelligence Improves Detection of Higher-Risk Eyes	Comparative case–control study of 339 eyes, 65 of which developed ectasia after LASIK (ectasia group) and 274 remained healthy (control group). Used 20-feature AI model. Reported that t-distributed stochastic neighbor embedding (t-SNE) visualization demonstrated greater ability to differentiate at-risk patients compared to all other methods used alone (*p* < 0.0001) [98].
[99]	2023	El-Naggar M.T. et al.	Assessment of Preoperative Risk Factors for Post-LASIK Ectasia Development	Retrospective case review which highlights that traditional topometric/tomographic parameters from Oculus Pentacam are insufficient for reliably predicting a large number of postoperative ectasia cases. For the 24 eyes from 15 patients which developed post-LASIK corneal ectasia, traditional indices such as the Ectasia Risk Score System (ERSS) measured more than 3 points in just 62.5% of cases, Belin/Ambrósio Enhanced Ectasia Display (BAD-D) deviation was higher than 1.6 in just 50% of cases and other indices were even lower. Only the AI-based Pentacam Random Forest Index (PRFI) reached more than 0.125 in 87.5% of cases [99].
[100]	2023	Francis M. et al.	New simulation software to predict postoperative corneal stiffness before laser vision correction	Study presenting the AcuSimX software (Narayana Ophthalmic Research and Development LLP, Bangalore, India—Full version AcuSimX, including the Inverse Finite Element Model (iFEM), Surgery-specific Finite Element Model (sFEM), and AI) and its use together with a decision tree AI classifier to better understand post-SMILE ectasia. The AI classifier exhibited a high AUC of 1.00, precision of 0.99 and recall of 0.99, for a threshold cutoff of 0.92 [100]. The authors reported a significant processing time of 1.47 h for the AcuSimX simulation on a medium-spec system (8 core 14 nm-generation CPU and 8 Gigabytes of RAM) [100].
AI LLMs and big data in patient selection for refractive surgery				
[101]	2024	Li J. et al.	Choice of refractive surgery types for myopia assisted by machine learning based on doctors’ surgical selection data	Very large single-center ML study on 7081 patients. Pre-analysis data curation using Synthetic Minority Over-Sampling Technique (SMOTE) using 18 years of patient data records. Evaluated the following six ML models: RF, Neural Basis Model (NBM), AdaBoost, XGBoost, backpropagation (BP) Neural Network (NN), Deep Belief Network (DBN) NN. Reported RF model had highest rate of agreement with ophthalmologist decisions as high as 0.8775 and Macro F1 as high as 0.8019. The model was able to provide confident recommendations for surgery type [101].
[102]	2020	Yoo T.K. et al.	Explainable Machine Learning Approach as Tool to Understand Factors Used to Select the Refractive Surgery Technique on the Expert Level	Multiclass XGBoost model to classify 18,480 patients (training 10,561, internal validation 2640, and external validation 5279) into the following four categories: laser epithelial keratomileusis, laser in situ keratomileusis, SMILE, and contraindication groups. The purpose was to select the surgery option. The multiclass XGBoost model exhibited an accuracy of 81.0% on internal and 78.9% on external validation datasets [102].
[103]	2019	Yoo T.K. et al.	Adopting machine learning to automatically identify candidate patients for corneal refractive surgery	Five heterogenous algorithms using large multi-instrument data and clinical decisions from experts to predict candidates for surgery. Subsequently, an ensemble classifier was developed to improve the performance. The study used 10,561 subjects for training, 2640 for internal validation, and 5279 for external validation. Reported AUC of 0.983 on internal validation and 0.972 on external validation. The ML models were superior to traditional percentage of tissue ablated and Randleman ectatic score [103].
[104]	2017	Achiron, A et al.	Predicting Refractive Surgery Outcome: Machine Learning Approach With Big Data	Very large single-center ML study on 17,592 cases that underwent photorefractive or LASIK surgery. Decision forest algorithm using 38 clinical parameters. Analysis returned efficacy of 0.7 or greater in 92% of cases and of 0.8 or greater in 84.9% of eyes. Factors correlated with decreased efficacy were age, central corneal thickness, mean keratometry, and preoperative Corrected Distance Visual Acuity (CDVA). Efficacy increased with pupil size [104].
Using AI as an educational tool in refractive surgery				
[105]	2026	Zhou, M. et al.	Comparing the performance of four mainstream large language models on medical literature review generation: a human expert evaluation in SMILE surgery	Study which used commercially available LLMs (OpenAI GPT4, Google Gemini 2.5 Pro, X Grok-3, and DeepSeek R1) to generate a literature review on nine topics related to SMILE. Reported that LLMs demonstrated significant performance variations, with models having diminished performance on open topics and exhibiting reference fabrication (“hallucinations”) [105].
[106]	2025	Wallerstein A. et al.	ChatGPT-4o and OpenAI-o1: A Comparative Analysis of Its Accuracy in Refractive Surgery	Comparative study of two OpenAI LLMs using a randomized questionnaire with 228 text-only questions from the American Association of Ophthalmology (AAO) Basic and Clinical Science Course (BCSC) on refractive surgery. Reported that OpenAI-o1 achieved the highest score 91.2%, ChatGPT-4o an intermediate score 86.4%, and 1983 ophthalmology residents achieved 77% [106].
[107]	2025	Attal L. et al.	Comparative Assessment of Large Language Models in Optics and Refractive Surgery: Performance on Multiple-Choice Questions	Comparatively evaluated seven LLMs in answering multiple-choice questions in optics and refractive surgery using 134 questions from national ophthalmology certification exams. Reported that Open AI ChatGPT O1 achieved the highest overall accuracy of 83.5%, excelling in complex optical calculations (84.1%) and optics questions (82.4%). Reported that DeepSeek V3 displayed the highest accuracy in refractive surgery-related questions (89.7%) followed by ChatGPT O3 Mini (88.4%). Reported that ChatGPTO1 demonstrated near-comparable accuracy for both calculated and non-calculated questions (84.1% versus 83.3%). Suggested that the results could have potential applications in exam preparation and medical training contexts [107].
[108]	2025	Demir S.	Investigating the role of large language models on questions about refractive surgery	Investigated the role of three LLMs (Open AI ChatGPT 4.0, Microsoft Copilot, and Google Gemini) in providing clinically accurate information on 25 questions about refractive surgery. Reported that the ChatGPT 4.0. score was significantly higher, but with less easy-to-read answers [108].
[109]	2024	Aydın F.O. et al.	Readability and Appropriateness of Responses Generated by ChatGPT 3.5, ChatGPT 4.0, Gemini, and Microsoft Copilot for FAQs in Refractive Surgery	Four-LLM study which evaluated responses to 40 frequently asked patient questions. Reported that Open AI ChatGPT 3.5 was correct in 45% of cases, ChatGPT 4.0 in 52.5%, Google Gemini in 87.5%, and Microsoft Copilot in 60% of cases. Reported that Google Gemini provided the most complete but difficult-to-read answers [109].
[110]	2024	Tuttle J.J. et al.	Learning the Randleman Criteria in Refractive Surgery: Utilizing ChatGPT 3.5. Versus Internet Search Engine	Comparative study of 23 medical students using Open AI ChatGPT 3.5. versus an internet search engine to define the Randleman criteria and its five parameters. Reported that ChatGPT provided the correct definition for only 26.1% of students, while using internet search students found the correct definition in 100% of cases. Reported ChatGPT 3.5. hallucinations such as “Randleman syndrome” or misidentifying Randleman criteria as a corneal ectasia staging system [110].
[111]	2024	Sensoy E. & Citirik M.	Exploring Artificial Intelligence Programs’ Understanding of Lens, Cataract, and Refractive Surgery Information	Study which evaluated three commercially available LLMs (OpenAI GPT, Microsoft Bing, and Google Bard) and their understanding of lens, cataract, and refractive surgery. Reported correct results to only 50% (ChatGPT), 61.4% (Bing), and 71.4% (Bard) of questions. Cautioned that LLMs’ understanding of refractive surgery is not fully accurate [111].
[112]	2024	Petroff, D. J. et al.	Evaluating the Accuracy of Artificial Intelligence (AI)-Generated Illustrations for Laser-Assisted In Situ Keratomileusis (LASIK), Photorefractive Keratectomy (PRK), and Small Incision Lenticule Extraction (SMILE)	Paper detailing patient education about refractive surgery using AI-generated illustrations. Used Decoder-Only Autoregressive Language and Image Synthesis 3 (DALL-E 3) and Medical Illustration Master (MiM). Images were generated using each AI model and rated versus human-expert-produced images. Reported that human experts outperformed AI-generated images. AI images presented vibrant illustrations; however, they were not sufficiently accurate or educational in design [112].
AI use in Intrastromal Corneal Ring surgery				
[113]	2026	Perez, E. et al.	Machine Learning Model for Predicting Visual Acuity Improvement After Intrastromal Corneal Ring Surgery in Patients With Keratoconus	Retrospective study on 120 eyes of 102 keratoconus patients with Intrastromal Corneal Ring (ICRS) implantations. Multiple AI models were trained to predict postoperative visual acuity improvements. Reported AI models demonstrated excellent performance, with XGBoost achieving a perfect result in predicting postoperative visual improvement [113].

Abbreviations: Ref. nr.—reference number; LASIK—Laser In Situ Keratomileusis; RF—Random Forest; AUC—Area Under the Curve; SILK—Smooth Incision Lenticule Keratomileusis; D—Diopter; LLM—Large Language Model; EOZ—Effective Optical Zone; SMILE—Small-Incision Lenticule Extraction; CCT—central corneal thickness; RST—Residual Stromal Thickness; LT—Lenticule Thickness; AACM-PP—Anterior Axial Curvature Map and Preoperative Parameter-Based Model; SE—Spherical Equivalent; ML—Machine Learning; AI—Artificial Intelligence; LM—linear model; UDVA—Uncorrected Distance Visual Acuity; HOAs—Higher-Order Aberrations; RTA—Relational Tissue Altered Index; PTA—Percent Tissue Altered; Grad-CAM—Gradient-weighted Class Activation Mapping; GAMM—Generalized Additive Mixed Model; NN—Neural Network; RMSE—Root Mean Square Error; Trans-PRK—Transepithelial Photorefractive Keratectomy; AS-OCT—Anterior Segment Optical Coherence Tomography; ERSS—Ectasia Risk Score System; BAD-D—Belin/Ambrósio Enhanced Ectasia Display; PRFI—Pentacam Random Forest Index; iFEM—Inverse Finite Element Model; sFEM—Surgery-specific Finite Element Model; SMOTE—Synthetic Minority Over-Sampling Technique; NBM—Neural Basis Model; BP—backpropagation; DBN—Deep Belief Network; CDVA—Corrected Distance Visual Acuity; DALL-E 3—Decoder-Only Autoregressive Language and Image Synthesis 3; MiM—Medical Illustration Master; ICRS—Intrastromal Corneal Ring; T-CAT—Topography-guided Custom Ablation Treatment.

**Table 3:** Original articles on keratoconus AI applications analyzed in this paper.

AI Keratoconus Management and Corneal Ectasia Screening for Refractive Surgery				
[114]	2026	Shetty, R. et al.	Corneal Tomography Changes After Simultaneous Topical Application of Cyclosporine and Trehalose in Keratoconus Evaluated with an Artificial Intelligence Model	Retrospective study using 724 Pentacam scans on 362 eyes (181 patients) with follow-up up to 36 months (mean 10.8 ± 6.01 months). The study focused on the clinical effect of the treatment; previously published AI models were used (Shetty R. et al. 2021) [115]. Reported clinical improvements from the treatment and recommended further studies [114].
[116]	2026	Wang, M. H., & Qin, S.	Explainable neuro-symbolic artificial intelligence for automated interpretation of corneal topography and early keratoconus detection	Prospective pilot cohort study on 20 eyes proposing an integrative framework for keratoconus detection using CNN-processed corneal maps and LMM-based report generation. The following multimodal approach was used: biometric parameter extraction from IOLMaster 700 via parsing pipeline; CNN Vision Transformer (CNN-ViT) corneal image processing; feature-to-knowledge embedding alignment module; and diagnostic reasoning using a probabilistic logic inference layer. Reported Area Under the Curve (AUC) of 0.95 and fast processing time of 95 ± 12 s per case [116].
[117]	2026	Patil, R. P. et al.	Advancing subclinical keratoconus detection using polarization-sensitive optical coherence tomography and artificial intelligence	Study combining Polarization-sensitive Optical Coherence Tomography (PS-OCT) and a Random Forest (RF) Artificial Intelligence (AI) classifier. Reported AUC of 0.91, 83% precision, and 82% accuracy. Reported better results for Pentacam imaging with an AUC of 0.95 and 86% precision and accuracy. For MS-39, the study reported an AUC of 0.94 and 86% precision and accuracy. Recommended PS-OCT as complementary method to Pentacam or MS-39 [117].
[118]	2025	Giap, B. D. et al.	Deep Learning-Based Eye Rubbing Detection Using Wrist-Based Wearable Devices to Enable Rigorous Study of Risk Factors for Ectasia Progression	Clinical study on eye rubbing as a risk factor for corneal ectasia progression. Used 6 Deep Learning (DL) architectures to interpret data from wrist-based wearable devices. Reported AUC of 98.26 ± 0.92% in detecting eye-rubbing versus normal activity and AUC 96.70% on testing set. The study thus proposed a novel avenue for eye rubbing detection which could be especially useful in monitoring pediatric corneal ectasia or keratoconus patients [118].
[119]	2025	Wang M. H.	An Explainable AI Framework for Corneal Imaging Interpretation and Refractive Surgery Decision Support	Prospective pilot study on 20 eyes combining LLMs with corneal topography data from a ZEISS IOL Master 700. AI backbones were as follows: computational pipeline using PyTorch 2.2.1 for DL model, Pyro 1.8 for Bayesian probabilistic reasoning, and HuggingFace Transformers 4.42 for LLM integration. Used CNN-ViT for image extraction and processing. Model achieved 0.95 ± 0.03 AUC, 92 ± 4% sensitivity, and 94 ± 5% specificity in corneal ectasia screening. For classifying eyes as suitable for refractive surgery, Wang M. H. reported 91% precision and 90% recall. Reported good feasibility for using Neuro-Symbolic + LLM framework in ophthalmological diagnosis [119].
[120]	2025	Du, K. et al.	Enhancing Early Keratoconus Detection With Multimodal Machine Learning: Integrating Tomography, Biomechanics, and Clinical Risk Factors	Large multi-cohort, multimodal ML study on 1035 eyes (1035 patients) which followed early keratoconus detection using Pentacam imaging and Corvis ST corneal hysteresis measurements. Evaluated six ML models. Most accurate model (CatBoost post-feature selection) for Forme Fruste Keratoconus (FFKC) achieved AUC 0.975. For subclinical keratoconus, it achieved an AUC of 0.991 and for clinical keratoconus, it achieve a perfect AUC of 1.00. Reported high success in using multimodal ML system integrating both tomography (Pentacam) and corneal biomechanics parameters (from Corvis ST). The study is thus novel in combining information from the two devices with higher comparative ML results versus only Pentacam imaging [120].
[121]	2024	Raju R.S. et al.	Validation of an Artificial Intelligence-based Tool—The Screening Corneal Objective Risk of Ectasia Integrated into Anterion for Detection of Corneal Ectasia/Risk of Ectasia	Prospective study including 320 eyes of 160 patients evaluated for refractive surgery and keratoconus using the Screening Corneal Objective Risk of Ectasia (SCORE) AI model. Reported the mean SCORE in normal eyes was −0.7 ± 0.6, 83.3% sensitivity, and 80% specificity. Reported SCORE specificity for keratoconus of 100% and 79% sensitivity [121].
[122]	2024	Drira I. et al.	Eye-Rubbing Detection Tool Using Artificial Intelligence on a Smartwatch in the Management of Keratoconus	Study which used gyroscope, accelerometer, and linear acceleration sensor data from commercial smartwatches to train two DL and four ML algorithms for detecting eye rubbing. The model achieved an accuracy of 94%, proving automated detection of eye rubbing is feasible for following patients at risk of corneal ectasia or keratoconus [122].
[123]	2024	Mourgues, E. et al.	Forme fruste keratoconus detection with OCT corneal topography using artificial intelligence algorithms	ML study using SS-OCT topography imaging from Tomey CASIA 2 and two ML models: RF and Logistic Regression (LR). Analyzed 108 keratoconus eyes, 88 FFKC eyes, and 162 normal corneas. Reported that LR and RF models detected FFKC with 0.99 and 0.98 AUC, respectively. Machine Learning (ML) model sensitivity was significantly better than the standardized Ectasia Screening Index (ESI) (LR 100% versus ESI 28%, RF 84% versus 28% ESI). Specificity was equal for LR ML model and ESI (100%) and lower for RF ML model (90%) [123].
[124]	2024	Quanchareonsap W. et al.	Deep Learning Algorithm for Keratoconus Detection from Tomographic Maps and Corneal Biomechanics: A Diagnostic Study	Pentacam (Oculus) and Corvis ST (Oculus) study using 1668 tomographic (769 patients) and 611 biomechanical (307 patients) images. Trained 3 AI models 1st with just Pentacam data, 2nd with combined Pentacam + Corvis ST biomechanical data, and 3rd with additional biomechanical index from Corvis ST. Reported AUC of 0.938 for Pentacam model and 0.985 for combined model. reported improvement in sensitivity for combined model (93% from 90.8%). Reported further improvements for model 3 using all 2nd model data + biomechanical index: AUC 0.991, accuracy of 95.6%, sensitivity of 93%, and specificity of 94.3%. Recommended combining corneal imaging data with biomechanical data [124].
[125]	2023	Kundu, G. et al.	Artificial intelligence-based stratification of demographic, ocular surface high-risk factors in progression of keratoconus	Prospective study with 450 keratoconus patients using a RF ML model to classify patients. The model correctly predicted 76% of progression cases and 67% of no progression cases based on first visit corneal parameters [125].
[126]	2023	Ambrósio, R. et al.	Optimized Artificial Intelligence for Enhanced Ectasia Detection Using Scheimpflug-Based Corneal Tomography and Biomechanical Data	Very large study on 3412 patients using Pentacam imaging and Oculus Corvis ST data to compare the Tomographic and Biomechanical Index (TBI) version 1 (TBIv1) versus AI-augmented TBI version 2 (TBIv2). AI model interpreted that TBIv2 had a higher AUC of 0.985 versus 0.974 for TBIv1. The study is important for clinically recommending AI optimization for Scheimpflug-based corneal tomography and biomechanical assessment [126].
[127]	2023	Kundu G. et al.	Universal architecture of corneal segmental tomography biomarkers for artificial intelligence-driven diagnosis of early keratoconus	Used imaging data from patients with stable, asymmetric or bilateral keratoconus using the MS-39 (CSO, Italy) machine. Reported AI model (Random Forest algorithm) achieved AUC of 0.994, accuracy of 95.6%, recall of 98.5%, and precision of 92.7% for detecting healthy eyes. Included keratoconus eyes reported AUC of 0.994, accuracy of 99.1%, recall of 98.7%, and precision of 99.1%. Furthermore, AI reclassified 36 Very Asymmetric Ectasia (VAE) eyes as healthy and efficiently classified VAE eyes as subclinical and forme fruste [127].
[128]	2022	Cohen E. et al.	Use of machine learning to achieve keratoconus detection skills of a corneal expert	Very large study using 8526 corneal tomography images of 4904 eyes using 2 ML RF algorithms. First ML algorithm included 94 instrument-derived parameters and the second ML model integrated both the parameters and the device-integrated keratoconus prediction indices. Reported that both RF algorithms were superior to tomographer’s standard keratoconus detection algorithms. First ML RF model had 90.2 accuracy, 94.2% sensitivity, and 89.6% specificity, the second model 91.5% accuracy, 94.7% sensitivity, and 89.8% specificity [128].
[129]	2022	Mohammadpour M. et al.	Comparison of Artificial Intelligence-Based Machine Learning Classifiers for Early Detection of Keratoconus	Prospective comparative study on 212 eyes of 212 patients using several ML classifiers. Reported highest agreement with the clinical diagnosis for Sirius Phoenix (sensitivity of 84.62%, specificity of 90.0%) followed by Pentacam Belin/Ambrósio Enhanced Ectasia Total Deviation Value (BADD) at 55.56% sensitivity and 86.08% specificity [129].
[130]	2022	Tan, Z. et al.	Artificial Intelligence-Based Diagnostic Model for Detecting Keratoconus Using Videos of Corneal Force Deformation	Study on 177 keratoconus eyes of 143 patients using a novel five-layer feed-forward (5-FNN) AI network. The study processed corneal deformation video frames, extracting contour data. The Highest Concavity Deformation Amplitude (HC-DA) and Radius at Highest Concavity (HC-R) were calculated. Four additional corneal parameters were added into the model. The 5-FNN AI network used backpropagation and three hidden layers of 20 neurons each. The 5-FNN model was trained on 276 samples before testing clinical results. Reported 5-FNN AI accuracy of 99.6% and 99.3% sensitivity on clinical data [130].
[115]	2021	Shetty R. et al.	Artificial Intelligence Efficiently Identifies Regional Differences in the Progression of Tomographic Parameters of Keratoconic Corneas	Retrospective study with 5 AI models using 1884 Pentacam images of 366 eyes. Reported AUC of 0.90–0.93, sensitivity of 85–89%, and specificity of 82–91% depending on the model. Concluded that AI models successfully identified eyes at risk with increases in Kmax and corresponding progression, thus correctly selecting eyes that may require earlier corneal crosslinking [115].
[58]	2020	Xie Y. et al.	Screening Candidates for Refractive Surgery With Corneal Tomographic-Based Deep Learning	Large study with 1385 patients using DL model to screen refractive surgery candidates for corneal ectasia or prior myopic postoperative corneas. AI model built on InceptionResNetV2 CNN architecture (PIRSS). Reported total detection accuracy of 94.7% on validation data set and 91.9% sensitivity and 98.7% specificity on detecting corneal ectasia. Model had 98.1% sensitivity and 100% specificity in detecting prior myopia corneal refractive surgery. Overall, PIRSS outperformed senior ophthalmologists in early keratoconus detection (PIRSS 95% versus 92.8% senior ophthalmologists) and significantly outperformed student refractive surgeons (PIRSS 95% versus 85.6%). Traditional Topographical Keratoconus Classification (TKC) behaved similarly to PIRSS in keratoconus cases; however, it only detected 4 out of 20 suspected images versus PIRSS. Overall, PIRSS was superior in detecting at-risk corneas versus TKC or senior ophthalmologist clinical assessment [58].
[131]	2019	Kamiya, K. et al.	Keratoconus detection using deep learning of color-coded maps with anterior segment optical coherence tomography: a diagnostic accuracy study	Study on 304 keratoconus eyes and 239 age-matched healthy eyes using DL model and Anterior Segment Optical Coherence Tomography (AS-OCT) corneal tomography images. Reported accuracy of 0.991; posterior elevation map showed highest accuracy of 0.993; and anterior curvature map showed lowest accuracy of 0.976. Reported 0.874 accuracy in classifying keratoconus stage. Concluded that DL using AS-OCT color-coded maps is highly effective in discriminating keratoconus from healthy corneas and can also classify keratoconus stage [131].
[132]	2017	Ruiz Hidalgo, I. et al.	Validation of an Objective Keratoconus Detection System Implemented in a Scheimpflug Tomographer and Comparison With Other Methods	ML study using 25 Pentacam-derived parameters to classify 131 eyes into keratoconus, keratoconus suspect, postrefractive surgery, and normal eyes. ML model, Keratoconus Assistant (KA), was benchmarked versus corneal specialists from two separate centers. KA agreed with 92.6% of keratoconus cases graded by specialists in the first center and 98% of cases graded from the second center. In keratoconus suspect and forme fruste detection, KA showed 65.2% agreement with specialists from one center and 100% with specialists from the second center. Concluded that KA is effective in detecting early keratoconus and an excellent assistant to trained clinical judgment [132].
[133]	2017	Ambrósio R. Jr. et al.	Integration of Scheimpflug-Based Corneal Tomography and Biomechanical Assessments for Enhancing Ectasia Detection	This landmark study introduced the Tomographic and Biomechanical Index (TBI), which is an AI tool for detecting ectasia that uses combined data from Oculus Pentacam corneal tomography and biomechanical data from the Oculus Corvis ST device (Oculus Optikgeräte GmbH, Wetzlar, Germany). The authors developed a Random Forest (RF) ML algorithm which achieved an AUC of 0.996 in detecting ectasia. TBI outperformed traditional factory-installed indices for keratoconus with statistically higher AUC (0.996) than the BAD-D AUC of 0.956 and Corvis Biomechanical Index (CBI) AUC of 0.936 [133].
[134]	2012	Arbelaez, M. C. et al.	Use of a support vector machine for keratoconus and subclinical keratoconus detection by topographic and tomographic data	Large study on 877 eyes with keratoconus, 426 eyes with subclinical keratoconus, 940 eyes with a history of corneal surgery (classified as abnormal), and 1259 healthy control eyes. The study used a Support Vector Machine (SVM) AI classifier. For abnormal classification, the SVM AI achieved 96% accuracy, 95.8% precision, 89.3 sensitivity, and 98.5% specificity. For keratoconus, the SVM AI achieved 96.9% accuracy, 94.6% precision, 92.8% sensitivity, and 98.2% specificity. For subclinical keratoconus, the SVM AI model achieved 93.3% accuracy, 57.4% precision, 75.2% sensitivity, and 94.9% specificity. For detecting normal eyes, the SVM AI mode achieved 94.9% accuracy, 93.7% precision, 93.1% sensitivity, and 96.0% specificity [134].
[135]	2010	Saad A. & Gatinel D.	Topographic and tomographic properties of forme fruste keratoconus corneas	Study on 143 eyes using the Nidek Corneal Navigator (Nidek, Gamagori, Japan) to train a computer Neural Network (NN) to recognize specific classifications of corneal topography. AI training images were acquired using two videokeratographs: Orbscan IIz (Bausch & Lomb Surgical, Rochester, NY) and OPD-Scan (Corneal Navigator, Nidek Co. Ltd., Gamagori, Japan). The study reported results focusing on distinguishing FFKC characteristics using a NN, identifying Thinnest Point (TP), Corneal Thickness Spatial Profile (CTSP), Percentage of Thickness Increase (PTI), and Percentage of Variation in Anterior (PVAK) as significantly different in FFKC versus normal groups (AUC 0.98) and between KC and normal groups (AUC 0.99) [135].
[136]	2005	Twa M.D. et al.	Automated decision tree classification of corneal shape	Comparative study of 132 normal eyes of 92 subjects and 112 eyes of 71 patients with keratoconus. A decision tree classifier was implemented via the C4.5 algorithm and compared to tomographer indices such as the Rabinowitz–McDonnell index, Schwiegerling Z3, Keratoconus Prediction Index (KPI), KISA%, Cone Location, and Magnitude Index. Reported 92% accuracy and AUC of 0.97 and reduction in information needed to distinguish between normal and keratoconus eyes using 4 of 36 Zernike polynomial coefficients [136].
[137]	2000	Chastang P.J. et al.	Automated keratoconus detection using the EyeSys videokeratoscope	Pioneering ML study on 208 corneas using a combination of index classifier + binary decision tree. First, the distribution of keratoconic and nonkeratoconic patterns was studied based on the value of each index from the videokeratoscope; this statistical analysis produced sensitivity and specificity curves based on index threshold. Afterwards, binary decision trees were built using the 6 indices (combined 2 at a time) with the highest sensitivity and specificity. Reported 88.5% sensitivity and 94.9% specificity in the validation data set. The authors reported that all statistical indices showed relatively low sensitivity and specificity so that alone they could not be used for keratoconus detection; the indices displayed high standard deviations. However, using the ML binary tree algorithms, this issue was resolved; to further improve sensitivity and specificity, the study authors hypothesized that combining more than 2 indices will be necessary. This study is notable as the first to use videokeratoscope data and ML for diagnosing keratoconus [137].
[138]	1995	Maeda N. et al.	Neural network classification of corneal topography. Preliminary demonstration.	Historical study which demonstrated the potential of NNs in interpreting corneal topography data. NN study on 183 topographic maps using human-classified ground truth (normal; with-the-rule astigmatism; mild, moderate, or advanced keratoconus; post-PRK; and post-keratoplasty). Used 108 maps for training set and 75 maps for test set. NN model integrated 11 topography indices and calculated data from a TMS-1 videokeratoscope. Reported that the NN correctly classified 60 of the 75 maps (80%) with specificity over 90% and very variable sensitivity (44% to 100%). The study is remarkable for pioneering NN applications in keratoconus [138].

Abbreviations: Ref. nr.—Reference number; AI—Artificial Intelligence; CNN—Convolutional Neural Network; LLM—Large Language Model; CNN-ViT—Convolutional Neural Network Vision Transformer; AUC—Area Under the Curve; PS-OCT—Polarization-sensitive Optical Coherence Tomography; DL—Deep Learning; IOL—Intraocular Lens; FFKC—Forme Fruste Keratoconus; SCORE—Screening Corneal Objective Risk of Ectasia; SS-OCT—Swept-Source Optical Coherence Tomography; LR—Logistic Regression; ML—Machine Learning; ESI—Ectasia Screening Index; TBI—Tomographic and Biomechanical Index; TBIv1—Tomographic and Biomechanical Index Version 1; TBIv2—Tomographic and Biomechanical Index Version 2; VAE—Very Asymmetric Ectasia; t-SNE—T-distributed stochastic neighbor embedding; 5-FNN—five-layer feed-forward; HC-DA—Highest Concavity Deformation Amplitude; HC-R—Radius at Highest Concavity; PIRSS—InceptionResNetV2; TKC—Topographical Keratoconus Classification; AS-OCT—Anterior Segment Optical Coherence Tomography; KA—Keratoconus Assistant; RF—Random Forest; BAD-D—Belin/Ambrósio Enhanced Ectasia Display; CBI—Corvis Biomechanical Index; SVM—Support Vector Machine; TP—Thinnest Point; CTSP—Corneal Thickness Spatial Profile; PTI—Percentage of Thickness Increase; PVAK—Percentage of Variation in Anterior; KPI—Keratoconus Prediction Index.

**Table 4:** Corneal ectasia and keratoconus screening performance of AI models.

AI	Article	Article Year	AUC	Accuracy	Sensitivity	Specificity
NN	Maeda N. et al. [138]	1995	-	80–100%	44–100%	80%
DT	Chastang P.J. et al. [137]	2000	-	-	88.5%	94.9%
DT	Twa M.D. et al. [136]	2005	0.97	92%	-	-
NN	Saad A. & Gatinel D. [125]	2010	0.98–0.99	-	-	-
SVM	Arbelaez M.C. et al. [134]	2012	-	96%, 96.9% & 94.9%	89.3%, 75.2% & 93.1%	98.5%, 94.9% & 96%
TBIv1	Ambrósio R. Jr. et al. [133]	2017	0.999 KC 0.899 VAE-NT	-	98.5% KC 76% VAE-NT	98.6% KC 89.1% VAE-NT
TBIv2	Ambrósio, R. et al. [126]	2023	0.999 KC 0.945 VAE-NT	-	98.7% KC 84.4% VAE-NT	99.2% KC 90.1% VAE-NT
ML	Ruiz Hidalgo, I. et al. [132]	2017	-	92.6–98%	-	-
DL	Kamiya, K. et al. [131]	2019	-	0.991	1.000	0.984
PIRSS (CNN)	Xie Y. et al. [58]	2020	0.993	94.7%	91.9%	98.7%
RF	Shetty, R. et al. [115]	2021	0.90–0.93	83–91%	85–89%	81–82%
5-FNN	Tan Z. et al. [130]	2022	-	99.6%	99.3%	100%
Sirius Phoenix	Mohammadpour M. et al. [129]	2022	-	80.02–96.60%	84.62%	90%
LightGBM DTB	Santhiago, M. R. et al. [98]	2022	0.99	-	-	-
RF	Cohen E. et al. [128]	2022	0.969	91.5%	94.7%	89.8%
RF	Kundu G. et al. [127]	2023	0.994	99.1%	-	-
RF	Kundu G. et al. [125]	2023	0.994	95.6%	-	-
EfficientNet-B7	Quanchareonsap W. et al. [124]	2024	0.985	0.956	93.0%	94.3%
LR & RF	Mourgues, E. et al. [123]	2024	0.99 LR 0.98 RF	0.988 LR 0.977 RF	100% LR 84% RF	100% LR 90% RF
SCORE	Raju R.S. et al. [121]	2024	-	-	83.3%	80%
6 ML: LR, RF, SVM, XGBoost, CatBoost, GBM	Du, K. et al. [120]	2025	0.976–1 (Internal) 0.952–1 (External)	0.922–1 (Internal) 0.881–0.919 (External)	0.917–1 (Internal) 0.762–1 (External)	0.923–1 (Internal) 0.892–0.951 (External)
LLM + DL + CNN-ViT	Wang M. H. [119]	2025	0.95 ± 0.03	93 ± 4%	92 ± 4%	94 ± 5%
RF	Patil, R. P. et al. [117]	2026	0.91 PS-OCT 0.95 Pentacam 0.94 MS-39	82% PS-OCT 86% Pentacam and MS-39	-	-
LLM + CNN-ViT	Wang, M. H., & Qin, S. [116]		0.95	93 ± 4	92 ± 4%	94 ± 5%
RF	Shetty, R. et al. [114]	2026	0.93 Kmax 0.75 D cutoff 0.96 Kmax 1.00 D cutoff 0.94 Kmax 1.25 D cutoff	-	-	-

AUC—Area Under the Curve; KC—Keratoconus; NN—Neural Network; DT—decision tree; TBIv1—Tomographic and Biomechanical Index Version 1; TBIv2—Tomographic and Biomechanical Index Version 2; ML—Machine Learning; DL—Deep Learning; PIRSS—InceptionResNetV2 Convolutional Neural Network (CNN) architecture; CNN—Convolutional Neural Network; RF—Random Forest; 5-FNN—five-layer feed-forward Neural Network; DTB—Decision Tree Boosting; LR—Logistic Regression; SCORE—Screening Corneal Objective Risk of Ectasia; SVM—Support Vector Machine; XGBoost—Extreme Gradient Boosting; CatBoost—Categorical Boosting; GBM—Light Gradient Boosting Machine; LLM—Large Language Model; CNN-ViT—Convolutional Neural Network Vision Transformer; PS-OCT—Polarization-sensitive Optical Coherence Tomography.

## Data Availability

For the purposes of this literature review, the presented data pertain to the original individual author’s papers, cited accordingly in the References section.

## Abbreviations

The following abbreviations are used in this manuscript:

PRK	Photorefractive Keratomileusis
FS-LASIK	Femtosecond Laser-Assisted In Situ Keratomileusis
Trans-PRK	Transepithelial Photorefractive Keratomileusis
SMILE	Small-Incision Lenticule Extraction
FLEx	Femtosecond Lenticule Extraction
ReLEx	Refractive Lenticule Extraction
CLEAR	Corneal Lenticule Extraction for Advanced Refractive Correction
RCLE	Refractive corneal lenticule extraction
SILK	Smooth Incision Lenticule Keratomileusis
AI	Artificial Intelligence
DL	Deep Learning
ARMD	Age-related Macular Degeneration
VRAM	Video Random Access Memory
RAM	Random Access Memory
CPU	Central Processing Unit
GPU	Graphics Processing Unit
CUDA	Compute Unified Device Architecture
NPU	Neural Processing Unit
APU	Accelerated Processing Unit
AMD	Advanced Micro Devices
WoS	Web of Science (Thomson Reuters)
PRISMA	The Preferred Reporting Items for Systemic Review and Meta-Analyses
ICL	Implantable Collamer Lens
RLE	Refractive Lens Exchange
EDOF	Extended Depth of Field
IOLs	Intraocular Lenses
SMILE	Small-Incision Lenticle Extraction
LLM	Large Language Model
ML	Machine Learning
CNN	Convolutional Neural Network
ViT	Vision Transformer
DR	Diabetic Retinopathy
AMD	Age-related Macular Degeneration
RTA	Relational Tissue Altered
PTA	Percent Tissue Altered
AS-OCT	Anterior Segment Optical Coherence Tomography
PIRSS	Pentacam InceptionResNetV2 Screening System
ICRS	Intracorneal Ring Segment
NN	Neural Network
SE	Spherical Equivalent
RTA	Relational Tissue Altered Metric
QUADAS-2	Cochrane Quality Assessment of Diagnostic Accuracy Studies
RF	Random Forest
NB	Naïve Bayes
AUC	Area Under the Curve
BAD-D	Belin/Ambrósio Enhanced Ectasia Display
D	Diopter
EOZ	Effective Optical Zone
CCT	Central Corneal Thickness
RST	Residual Stromal Thickness
LT	Lenticule Thickness
AACM-PP	Anterior Axial Curvature Map and Preoperative Parameter-Based Model
UDVA	Uncorrected Distance Visual Acuity
HOAs	Higher-Order Aberrations
Grad-CAM	Gradient-weighted Class Activation Mapping
GAMM	Generalized Additive Mixed Model
RMSE	Root Mean Square Error
SMOTE	Synthetic Minority Over-Sampling Technique
NBM	Neural Basis Model
BP	Backpropagation
DBN	Deep Belief Network
CDVA	Corrected Distance Visual Acuity
DALL-E 3	Decoder-Only Autoregressive Language and Image Synthesis 3
MiM	Medical Illustration Master
CNN-ViT	Convolutional Neural Network Vision Transformer
PS-OCT	Polarization-sensitive Optical Coherence Tomography
FFKC	Forme Fruste Keratoconus
SCORE	Screening Corneal Objective Risk of Ectasia
SS-OCT	Swept-Source Optical Coherence Tomography
LR	Logistic Regression
ESI	Ectasia Screening Index
TBI	Tomographic and Biomechanical Index
TBIv1	Tomographic and Biomechanical Index Version 1
TBIv2	Tomographic and Biomechanical Index Version 2
VAE	Very Asymmetric Ectasia
VAE-E	Very Asymmetric Ectasia Eye
VAE-NT	Fellow Eye of VAE-E with Normal Topography
KC	Keratoconus
t-SNE	T-distributed stochastic neighbor embedding
5-FNN	Five-layer feed-forward
HC-DA	Highest Concavity Deformation Amplitude
HC-R	Radius at Highest Concavity
TKC	Topographical Keratoconus Classification
KA	Keratoconus Assistant
GBDT	Gradient-Boosted Decision Tree
GAN	Generative Adversarial Network
SVM	Support Vector Machine
NNs	Neural Networks
1D	Unidimensional
2D	Two-dimensional
3D	Three-dimensional
AF	Activation Function
ReLU	Rectified Linear Unit
UWFP	Ultra-Widefield Fundus Photography
TFP	Traditional Fundus Photography
RLAIF	Reinforcement Learning from AI Feedback
RLHF	Reinforcement Learning from Human Feedback
API	Application Programming Interfaces
XGBoost	Extreme Gradient Boosting
DTB	Decision Tree Boosting
CatBoost	Categorical Boosting
GBM	(Light) Gradient Boosting Machine
TP	Thinnest Point
CTSP	Corneal Thickness Spatial Profile
PTI	Percentage of Thickness Increase
PVAK	Percentage of Variation of Anterior
KPI	Keratoconus Prediction Index
KNN	K-Nearest Neighbor
MRSE	Manifest Refraction Spherical Equivalent
GBR	Gradient-Boosting Regression
MAE	Mean Absolute Error
RSB	Residual Stromal Bed
µm	Micron
LogMAR	Logarithm of the Minimum Angle of Resolution
LINR	Linear Regression
BAGB	Bagging Aggregator Gradient Boosted
VMLP	Vanilla Multilayer Perceptron
BDMH	Bayesian Dynamic MultiHead Neural Network
OBL	Opaque Bubble Layer
BS	Black Spots
LS	Loss of Suction
ERSS	Ectasia Risk Score System
AWV	Age Weighted Value
T-CAT	Custom Ablation Treatment
FAQ	Frequently Asked Question
ART	Ambrósio’s relational thickness
PRFI	Pentacam Random Forest Index
iFEM	Inverse Finite Element Model
sFEM	Surgery-specific Finite Element Model
Kc_mean_	Postoperative corneal stiffness
N	Newton
m	Meter
mm	millimeter
DA_max_	Maximum Deformation Amplitude
nm	nanometers
IoMT	Internet of Medical Things
RVO	Retinal Vein Occlusion
